# Trapped by climate change? (In)voluntary immobility in Bangladesh

**DOI:** 10.1007/s10113-025-02452-3

**Published:** 2025-09-08

**Authors:** Jan Freihardt

**Affiliations:** Center for Comparative and International Studies (CIS), ETH Zurich, Haldeneggsteig 4, 8092 Zurich, Switzerland

**Keywords:** Environmental migration, Trapped populations, Involuntary immobility, Voluntary immobility, Riverbank erosion, Bangladesh

## Abstract

While there has been a considerable conceptual discussion of trapped populations in the environmental migration literature, the empirical evidence on their existence is scarce. I examine why many people remain in environmentally vulnerable areas by analyzing newly collected survey panel data of 1515 household heads living along the Jamuna River in Bangladesh, an area affected by riverbank erosion and flooding. To examine whether immobility is voluntary or involuntary, I assess the migration aspirations and capability to move of those who did not migrate after environmental shocks occurred in their village. The majority (82%) of non-migrants can be classified as voluntary non-migrants, while 13% can be considered “trapped” and the remaining 5% as acquiescent non-migrants. Being affected by erosion significantly increases the likelihood that immobility is involuntary by 7.9 percentage points, while also lowering the socio-economic status of the affected population. Taken together, these results suggest that environmental change may indeed lead to the “trapping” of parts of the population in exposed areas by eroding their capability to move while simultaneously increasing their migration aspirations. The findings have important policy implications, as they raise a word of caution against prematurely labeling all populations staying in environmentally exposed areas as “trapped” without considering their migration aspirations—a majority may in fact be voluntary non-migrants.

**Supplementary Information:**

The online version contains supplementary material available at 10.1007/s10113-025-02452-3.

## Introduction

Global warming increases the frequency and intensity of extreme weather and climate events such as cyclones, floods, heatwaves, droughts, and sea level rise (IPCC 2018; 2021). These climate events threaten the lives and livelihoods of millions of people around the world. Intuitively, we might expect that people living in areas highly exposed to climate risks would migrate to less exposed areas. And yet, various studies suggest that most people remain in their place of residence even when environmental conditions deteriorate dramatically (Gray and Mueller 2012; Jamero et al. 2017; Khatun et al. 2022; Mallick and Mallick 2021; Penning-Rowsell et al. 2013). This leaves us with a puzzle: Why do so many people continue to stay in highly exposed areas? Is it because they want to stay or because they cannot leave?

Previous studies have emphasized the notion of “trapped populations” to describe those parts of a population that would like to move away from their current location, but that lack the capability to do so. Hence, they might get trapped in environmental conditions that threaten their homes, livelihoods, and/or lives (Black et al. 2013; Foresight 2011; Ekoh et al. 2023). A second strand of literature argues that economic reasons are not the only explanation for why people stay put—we also need to consider non-material aspects such as geographical, socio-psychological, and affective factors (Adams 2016; Schewel 2020).

While there has been a considerable conceptual discussion of trapped populations in the environmental migration literature (see Ayeb-Karlsson et al. 2018 for a discursive review), the empirical evidence on their existence is scarce. At the macro-level, Nawrotzki and DeWaard (2018) provide evidence on potentially trapped populations in Zambia by combining high-resolution climate information with aggregated census data. At the micro-level, Koubi et al. (2022) examine how the adaptive capacity of affected individuals mediates the relationship between environmental change and migration behavior in five Majority World countries. Khatun et al. (2022) and Ahsan et al. (2022) identify involuntary non-migrants in Bangladesh using retrospective, self-reported survey data, which run the risk of motivated reasoning (Kunda 1990). However, none of these studies consider the respondents’ migration aspirations, meaning whether they would prefer to leave or stay. Without knowing people’s aspirations, however, inferences about trapped populations rely on strong assumptions that have not been rigorously tested, namely that *all* people who stay in the affected regions actually *want* to leave. In other words: It is impossible to understand whether the observed immobility^1^ is involuntary (as the term “trapped populations” suggests) or voluntary.

In this paper, I contribute to the understanding and measurement of environmental immobility. Specifically, this paper improves on the existing literature by explicitly considering the migration aspirations of respondents. In particular, I assess the pre- and post-monsoon migration aspirations of a population that stays put after riverbank erosion and flooding have occurred in their village. This allows me to identify those who would like to move but stay after experiencing environmental shocks. Hence, I can examine the existence, quantitative relevance, and socio-economic characteristics of trapped populations more directly than previous studies that do not consider migration aspirations at all (e.g., Koubi et al. 2022; Nawrotzki and DeWaard 2018), only qualitatively (Garcia et al. 2025), or only cross-sectionally (Mallick et al. 2023). Second, I examine why others actually *want* to stay (voluntary non-migrants), which is important information for policymakers aiming to reduce the vulnerability of communities living in exposed areas.

Empirically, I use newly collected individual-level panel data (*N* = 1515 randomly selected household heads living in 36 villages along 250 km of the Jamuna River in Bangladesh). Communities along the Jamuna are affected by riverbank erosion and flooding during the annual monsoon season. In selecting survey sites, I use a novel quasi-experimental design to compare affected and unaffected populations with similar baseline risk of experiencing the two types of environmental events. Respondents were randomly selected to be population representative of the entire eastern riverbank population based on an extensive pre-registered pre-analysis plan (see Appendix D). In addition to the environmental variables of interest, I collected individual-level information on the “capability to move” of those affected, including financial (i.e., socio-economic status) and human capital (i.e., education).

The results suggest that only 13% of those respondents who remain immobile following riverbank erosion and flooding can be classified as “involuntary non-migrants,” while 82% are “voluntary” and the remaining 5% are “acquiescent non-migrants.” Importantly, having been affected by erosion significantly increases the likelihood of immobility being involuntary. At the same time, erosion lowers the socio-economic status of affected households, suggesting that environmental shocks may indeed lead to involuntary immobility by undermining the capability to move of affected populations while increasing their migration aspirations. Among the self-reported reasons for staying near the river, place attachment, limited land availability elsewhere, and resource constraints are the most important factors. Overall, these findings have important policy implications, as they caution against prematurely labeling all populations remaining in environmentally vulnerable areas as “trapped”—a majority may in fact be voluntary non-migrants. It is therefore essential to take into account their migration aspirations and to consider the plethora of nuanced reasons why people remain in vulnerable areas—of which economic barriers are only one of many.

**Fig. 1:**
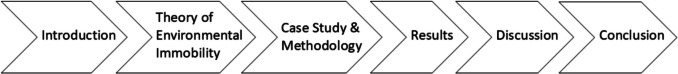
Structure of the article

The study’s limitations include its focus on individual-level data that overlooks household and community dynamics in migration decisions, its short-term scope limited to one monsoon season, the exclusion of political and institutional influences on (im)mobility, and the challenge of generalizing findings from a highly specific case study in the Jamuna River region to other contexts. The remainder of the article is structured as shown in Fig. 1.

## Immobility in the face of environmental vulnerability

Migration studies have long suffered from a mobility bias, focusing heavily on the drivers of migration and neglecting the reasons why many people do not migrate (Boas et al. 2019; Schewel 2020). It is only in recent years that immobility—understood as “spatial continuity in an individual’s center of gravity over a period of time” (Schewel 2020, 329)—has received increasing attention. Most notably, the UK government’s Foresight Report introduced the concept of “trapped populations” to describe people who are exposed to environmental risks but lack the capability to move away from these risks (Black et al. 2013; Foresight 2011). Subsequently, such involuntary immobility has dominated the discourse on environmental immobility, while voluntary immobility has received much less attention (Mallick and Schanze 2020; Schewel 2020).

I examine the micro-level processes underlying (both voluntary and involuntary) immobility in the context of environmental change. Theoretically, I base my argument on the aspirations-capabilities framework: Individuals will migrate if they have migration aspirations and at the same time have the capability to move (Carling 2002; Carling and Schewel 2018; de Haas 2021). Migration aspirations are “a general evaluation of whether or not migrating would be better than staying” (Carling and Schewel 2018, 948). Capability, on the other hand, refers to the “freedom to choose where to live, including the option to stay” (de Haas 2021, 2). Different dimensions can influence this freedom. For instance, capability may be positively associated with socio-economic status (Afifi et al. 2016; Black et al. 2013; Mallick and Mallick 2021), education (Rofi et al. 2006; Schewel and Fransen 2022), or—in contexts where strong social gender norms prevail—male gender (Akter et al. 2019; Mata-Codesal 2015; Pedraza 1991; Tripathy Furlong et al. 2022). Table 1 provides an overview of how these two key concepts relate to different (im)mobility outcomes.

**Table 1:** Aspirations-capabilities-derived individual (im)mobility types. Table based on de Haas (2021, 22)

		**Migration capabilities**	
		Low	High
Migration aspirations	High	Involuntary immobility (trapped populations)	Voluntary mobility
	Low	Acquiescent immobility	Voluntary immobility

The distinction between aspirations and capabilities is important for understanding environmental immobility. For example, Black et al. (2013) only consider an individual’s vulnerability to extreme events and their ability to move to infer the (conceptual) existence of trapped populations. However, this concept lacks an individual’s migration aspirations, which are crucial for identifying trapped populations: Even if people are exposed to environmental stress and unable to move, they may still prefer to stay in situ rather than move—in which case they would not be considered trapped populations. I therefore use migration aspirations to distinguish between voluntary, acquiescent, and involuntary immobility:

- Involuntary non-migrants (trapped populations): Individuals who would like to move, but cannot, and therefore remain where they are.
- Voluntary non-migrants: Individuals who stay put, preferring to stay rather than move, while having the capability to move.
- Acquiescent non-migrants: Individuals who stay put, and prefer staying to moving, but lack the capability to move.

**Fig. 2:**
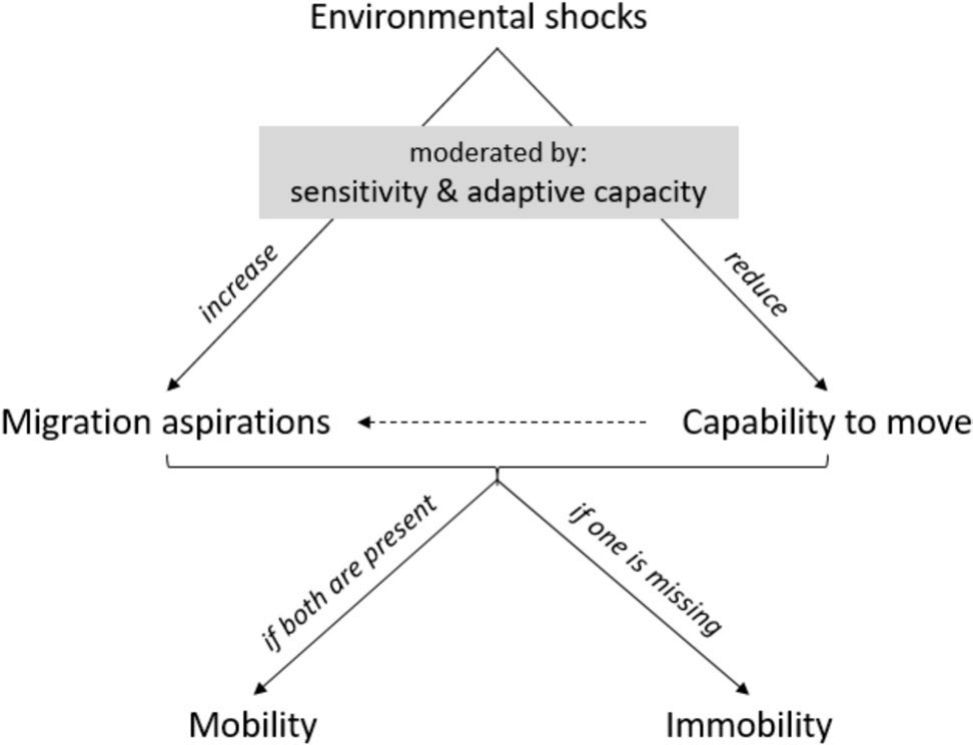
Impact of environmental shocks on migration aspirations and the capability to move. The dashed arrow indicates potential interactions between capability and aspirations

Next, as I examine immobility in the context of environmental shocks,^2^ I discuss the influence of such shocks on aspirations and capabilities (Fig. 2), drawing on a large literature suggesting that environmental shocks can act as “push” factors. First, exposure to environmental shocks may *increase* migration aspirations. This could be the case if the shock destroys a household’s home, leading to a desire to move elsewhere rather than rebuild in situ. Similarly, a shock may threaten a household’s livelihood opportunities, making a move to a place with better livelihood opportunities seem desirable. Indeed, environmental shocks have been found to increase migration aspirations (Bertoli et al. 2021; Helbling et al. 2021; Rudolph et al. 2025). Second, environmental shocks can *reduce* the capability to move. For example, they can affect a household’s socio-economic status by destroying assets (e.g., eroding land or killing livestock) or reducing disposable income (e.g., causing crop failure or limiting employment opportunities) and/or income opportunities. Evidence from different contexts suggests that environmental shocks have particularly severe effects on poor households, potentially leading to poverty traps (Arslan et al. 2016; Asfaw et al. 2015; Carter et al. 2006). Taken together, these two effects lead to my main hypothesis:

- H1: Environmental shocks can lead to involuntary immobility (trapped populations) by increasing migration aspirations while reducing the capability to move of affected populations.

These main effects of environmental shocks on aspirations and capabilities are moderated by a household’s sensitivity^3^ (“the degree to which a system^4^ is affected, either adversely or favorably, by climate variability or change,” IPCC 2007, 881) and adaptive capacity (“the ability of a system to adjust to climate change (…) to moderate potential damages, to take advantage of opportunities, or to cope with the consequences,” IPCC 2007, 21). Finally, capabilities can also influence aspirations: A high capability to move could reduce migration aspirations, for example, if high socio-economic status increases place attachment (e.g., through land ownership). Conversely, a low capability may also reduce migration aspirations—for example, a lack of education that could facilitate integration into the labor force elsewhere may reduce the attractiveness of moving and thus affect aspirations.

Such an interaction between aspirations and capabilities, as well as the moderating effects of sensitivity and adaptive capacity (which may themselves be related to aspects of the capability to move) can potentially complicate the analysis of environmental immobility considerably. Note, however, that these are second-order effects of lesser expected magnitude compared to the first-order, direct effects of environmental shocks on aspirations and capabilities, respectively. The following analysis will therefore focus on these first-order effects, while providing complementary evidence on the magnitude of some of the second-order correlations.

While the concept of trapped populations puts a strong focus on economic barriers to migration, recent work has highlighted that these are only one dimension of a plethora of reasons for immobility (Adams 2016; Schewel 2020). In particular, according to Schewel (2020), low migration aspirations (which can be related to both *voluntary* and *acquiescent* immobility, see Table 1) can be explained by (a) factors that “retain” (attractive conditions at the place of residence that increase the preference to stay, such as economic, social, or emotional ties); (b) factors that “repel” (unattractive conditions in a potential migration destination that reduce migration aspirations, such as perceived insecurity or a lack of a supportive migrant network); and (c) “internal constraints on decision-making” (such as risk aversion or internalized social norms). Accordingly, I argue that:

- H2a: People who prefer to stay (voluntary or acquiescent non-migrants) show, on average, higher levels of place attachment than those who prefer to move (involuntary non-migrants).
- H2b: People who prefer to stay (voluntary or acquiescent non-migrants) have, on average, less access to a migrant network than those who prefer to move (involuntary non-migrants).
- H2c: People who prefer to stay (voluntary or acquiescent non-migrants) show, on average, higher levels of risk aversion than those who prefer to move (involuntary non-migrants).

## Research design

### The case: Jamuna River in Bangladesh

Bangladesh is a prime case for studying environmental (im)mobility. Migration plays an important role in the country’s culture. In terms of international migration, Bangladesh is the sixth largest migrant-sending country in the world, with over seven million Bangladeshi migrants living abroad (IOM 2021). Internal migration is also an important income diversification strategy for many households. Migration rates that have been observed in different natural hazard-prone areas range from 36% for seasonal migration (Bryan et al. 2014), over 40% for at least one permanent move in the lifetime of respondents among the eastern riverbank population of the Jamuna—the area of this study—(Ferdous et al. 2018), to 95% for temporary migration during the flood season (Paul and Routray 2010). In terms of future migration flows, the World Bank’s Groundswell report estimates that up to 13.3 million people could be internally displaced within Bangladesh by 2050 due to climate change (Rigaud et al. 2018).

In terms of environmental change, Bangladesh is one of the countries most vulnerable to the adverse effects of climate change, due to its topography and its location in one of the largest river deltas in the world (Rigaud et al. 2018). It is severely affected by sea level rise, frequent cyclones, and heavy monsoon rains that increase river flows, which in turn contribute to widespread flooding and riverbank erosion (Hasan et al. 2018; Islam et al. 2021). All of these environmental events are projected to intensify in the future (IPCC 2018; 2021), adversely affecting people and their livelihoods by damaging their homesteads and agricultural land, and potentially causing economic and social disruptions leading to large migration flows (Freihardt 2025; Rigaud et al. 2018). In Bangladesh, riverbank erosion and flooding are the most impactful processes in terms of annual damage (Ahmed 2015).

In terms of riverbank erosion, about 20 out of 64 districts in the country are prone to erosion, which consumes about 8700 ha of land every year, affecting about 200,000 people (Alam 2017). Although communities along the rivers are aware of the erosion risks, people choose to settle along the rivers due to the high soil fertility and/or lack of other suitable space given the country’s high population density. Erosion has multiple negative impacts on affected communities, including the destruction of farmland, homes, and infrastructure such as roads and schools. Along the 250 km long Jamuna River, the case region of this study, the cumulative net erosion was approximately 933 km^2^ between 1973 and 2017 (CEGIS 2018). This would be equivalent to widening the river by more than 4 km if the erosion were evenly distributed along the river. In certain areas, erosion causes the riverbank to shift by several hundred meters per year, often for several years in a row. Erosion events occur mainly during the rainy monsoon season, typically from June to October. As the river erodes more soil than it can transport, some of the eroded soil is deposited downstream, forming new land in the form of islands (so called *chars*). However, the river erodes about seven times more land than it creates (Sarker et al. 2014).

In terms of flooding, it also occurs mainly during the monsoon, when the water level of the Jamuna River rises to the point where most of the land—and therefore most of the villages—on its riverbanks are inundated. This regular flooding plays an important role in the livelihood cycle of the riverine people, providing moisture and nutrients for their agricultural plots. It is only when a flood becomes too severe—either because the water rises too high or because it stays too long—that it can turn into a disaster, damaging crops, homes, or infrastructure (Alam 1990). Riverbank erosion and flooding are closely linked, as severe erosion typically occurs when the force of the flowing water increases just before and during the monsoon season.

The impacts of flooding and erosion are not uniform along and across the river, but vary according to local natural and anthropogenic characteristics such as elevation or embankments (Ferdous et al. 2018). These characteristics vary not only in space but also in time, as the Jamuna River is one of the fastest widening and changing river systems in the world (Oberhagemann, Haque, and Thompson 2020). Finally, there are also differences between households in their ability to cope with environmental change. People living along rivers have developed a range of coping strategies to minimize damage from flooding (e.g., raising the platform of the house; Paul and Routray 2010) and erosion (e.g., planting vegetation on the riverbank; Mamun et al. 2022). As coping strategies tend to be related to socio-economic status, not all households are equally able to adopt these strategies. This significant variation both between and within villages forms the basis of the research design used in this study, which is presented in the following subsection.

### Survey overview

**Fig. 3:**
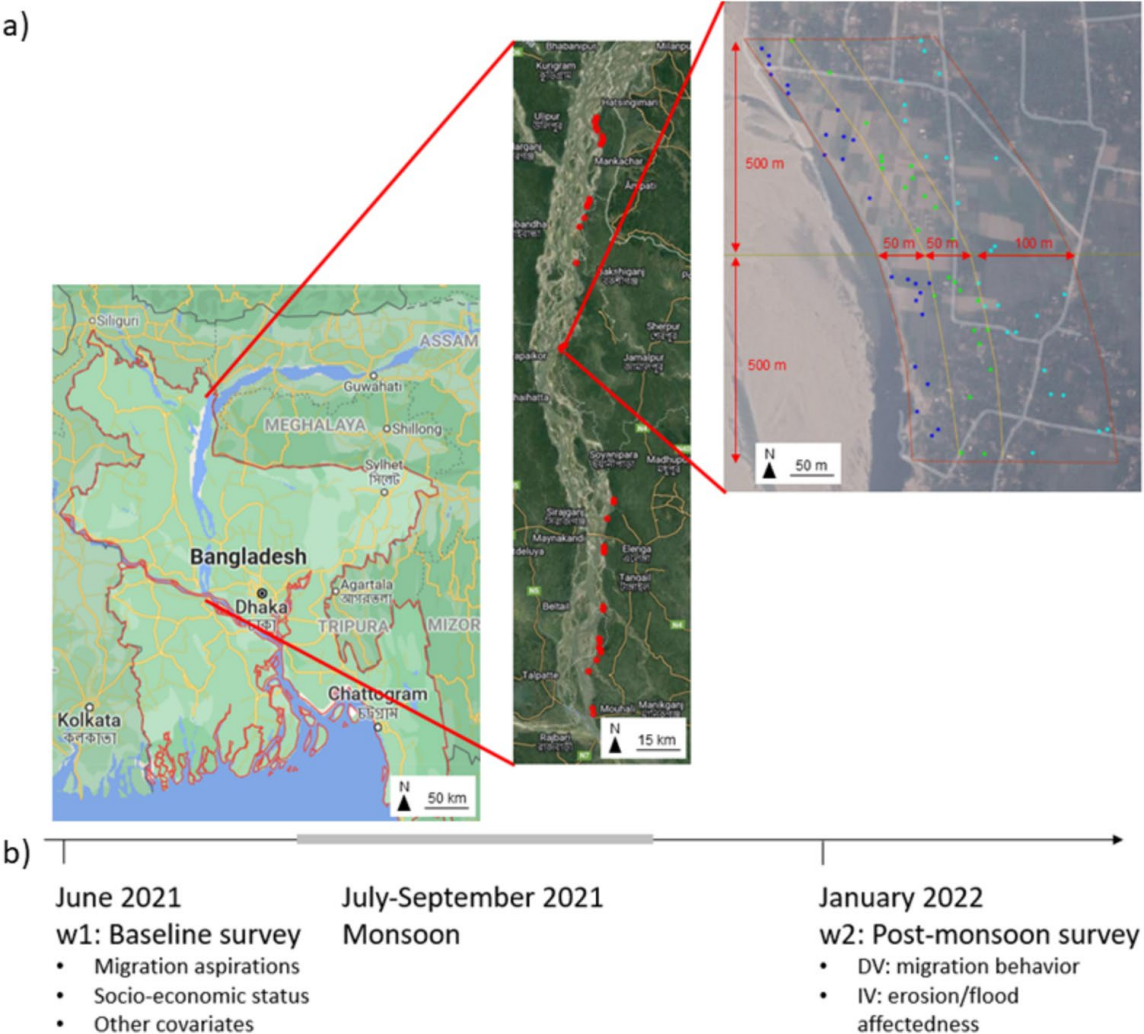
**a** Overview of the Jamuna River, the 36 study locations, as well as the three sampling zones within each location. Copyright map: Google. Copyright satellite image: TerraMetrics, 2022. **b** Timeline of survey waves and the monsoon season, and overview of which variables were assessed in which wave. DV: dependent variable. IV: independent variable

For the empirical analysis, I use data from two waves of a panel survey of 1515 household heads from 36 locations distributed along the entire length of the Jamuna River in Bangladesh (Fig. 3a). Participants were selected using a multi-stage cluster design, making the sample population representative of the riverbank population. In the first stage, all survey sites (1 km sections) potentially at risk of riverbank erosion along the easternmost bank line of the Jamuna River were identified. Of all the potential sites, 36 (86%) could be visited. At each of these 36 locations, households were sampled using a stratified random spatial sampling design to survey households located within three zones defined by distance from the riverbank (see Appendix A for further details on the selection procedure). I am therefore confident that the respondents represent a high quality sample^5^ of the riverbank population at risk of erosion and flooding in Bangladesh.

The baseline survey (wave w1) was conducted in June/July 2021, before the onset of the monsoon season, when flooding and erosion occur. The post-monsoon survey (wave w2) was conducted between January and March 2022 (Fig. 3b). In this wave w2, both non-migrants and migrants were re-interviewed. To track migrants, phone numbers were collected in wave w1, making it possible to contact and locate respondents even if they had left the village. Panel attrition between the two waves was less than 5% for those who had not migrated between waves w1 and w2 (Table S 4).

The respondents are 88% male, 92% married, 47 years old on average, and mostly have no or only primary education (Table S 5). Fifty-six percent of the respondents depend on the environment as their main source of income, either by working on their own or other people’s agricultural land. Other common sources of income include owning a small business/shop, non-agricultural day labor, remittances, transport, and textile weaving.

Interviews were conducted face-to-face in Bangla by local interviewers and lasted approximately 45–60 min. The questionnaire included both closed and open-ended questions on respondents’ experiences of migration and environmental events, as well as personal and household information. Respondents received monetary compensation for their time spent on the survey (100–150 Taka,^6^ equivalent to $0.92–1.38, or 50–80% of a day laborer’s daily wage). The study was pre-registered with OSF; details are provided in Appendix D.

### Operationalization

As introduced in the “Immobility in the face of environmental vulnerability” section, I define voluntary and involuntary immobility in the face of environmental change through an individual’s immobility status, their migration aspirations, and their capability to move (see also the operationalization strategy suggested by DeWaard et al. 2022). First, respondents’ *immobility* was assessed after the monsoon (see Fig. 3b for an overview of which variables are taken from which survey wave). Respondents were classified as non-migrants if they were living in the same village in wave w2 as in wave w1. In contrast, they were classified as migrants if they lived permanently (i.e., with the entire household) in a place outside the administrative boundaries of their original village.

Second, I measure the respondents’ *migration aspirations* in waves w1 and w2, taking a value of 1 if they either answered “Move somewhere else” to the question “Right now, if you could choose, would you stay here in this village or would you prefer to move to another place?” or “Yes” to the question “During the last month, have you thought seriously about leaving this village?”.^7^

Third, I examine three factors that might influence people’s *capability to move*. *Socio-economic status* is operationalized by the first component of a principal component analysis (PCA) on asset ownership, assessed in waves w1 and w2 (see Table S 6 for dimensions/components of the PCA). *Education* and *sex* were measured in wave w1.

*Environmental shocks*^8^ are operationalized by the respondents’ self-reported affectedness by erosion or flooding, respectively, during the 2021 monsoon season. This affectedness was assessed retrospectively in wave w2, by asking “Has the 2021 erosion (flood) had an impact on your household?” as well as —for those who had been affected—“What was the impact?” (see Table S 7 for an overview of the impact categories). In the analyses, I use binary indicators of affectedness, taking a value of 1 if a respondent indicated that their household had been affected by erosion or flooding, respectively.

While environmental perceptions have been shown to be important for understanding migration behavior (Koubi et al. 2016), using self-reported data to operationalize affectedness by environmental shocks may raise concerns about motivated reasoning: Respondents who have migrated might exaggerate the impact of erosion/flooding to justify their migration behavior (and vice versa).^9^ To address this concern, I compare respondents’ subjective assessment of the specific impact category “loss of house” with objective data. Specifically, I use respondents’ house coordinates as recorded during wave w1 and the satellite-based erosion assessment tool developed by Freihardt and Frey (2023) to identify those respondents whose house was eroded during the 2021 monsoon. Overall, I find that self-reported and objective data are consistent for 94% of all respondents, increasing my confidence in relying on self-reported impacts for the main analyses. A more detailed discussion can be found in Appendix B.

Several covariates (measured in wave w1) for which previous studies have found correlations with (im)mobility were included in the analyses: With regard to *occupation type*, I recoded the respondents’ main source of income as either environmentally independent (0) or dependent (1). In doing so, I assume that environmentally dependent respondents are more likely to migrate following an environmental event due to the strong link between their livelihoods and the environmental conditions (de Longueville et al. 2020). *Place attachment* is measured on a 5-point scale from 1- “not attached at all” to 5- “very attached.” It has been shown that place attachment can have a stronger effect on immobility than resource constraints (Adams 2016). Respondents self-reported their *risk preference* on a 5-point scale from 1- “I never take chances” to 5- “I always take chances.” Risk averse individuals are expected to be less likely to move, as the risk of moving is perceived to be higher than the risk of staying (Carling 2002). Respondents’ *migrant network* ranges from 1- “Almost all relatives in the same village” to 5- “Almost all relatives in other places in Bangladesh.” I expect respondents with larger migrant networks to be more likely to move due to increased support in potential destination areas (Zhao 2003). Other covariates included in the analysis were the respondents’ age and marital status, as these have been shown to influence migration behavior (Adger et al. 2015; Hunter et al. 2015). Summary statistics and a correlation matrix of all relevant variables are presented in Table S 5 and Table S 8, respectively. Overall, the variables are only weakly correlated, except for sex and marital status, where female sex is correlated with unmarried status.

### Estimation strategy

To compare voluntary/acquiescent and involuntary non-migrants, I estimate logistic regression models. The first set of models estimates only the direct effects of (a) erosion/flood affectedness and (b) the capability to move. A third model includes both affectedness and capability to test whether environmental shocks lead to involuntary immobility by reducing the capability to move. To test the robustness of the results, a final model adds the covariates introduced above as well as village-level fixed effects. All models include standard errors clustered at the village level.

To test the hypothesized effects of environmental shocks on migration aspirations and the capability to move, I use a difference-in-differences approach, with treatment corresponding to erosion and flood affectedness, respectively. As outcome variables, migration aspirations and socio-economic status (as a proxy for the capability to move) were measured in waves w1 and w2, respectively.

## Results

### Existence and quantification of voluntary and involuntary immobility in the face of environmental changes

I identify voluntary and involuntary non-migrants by examining the migration aspirations of those respondents who did not leave the village between waves w1 and w2 (N_non-migrants_ = 1456 out of N_total_ = 1515, corresponding to 96% non-migrants and 4% permanent migrants). Of these 1456 non-migrants, 193 (13% of the non-migrants) expressed aspirations to permanently leave the village in wave w2 (Table 2). When asked why they wanted to leave, respondents overwhelmingly cited erosion and flooding as the main reasons (Fig. S 3). As I argued in the “Immobility in the face of environmental vulnerability” section that migration occurs when migration aspirations coincide with the capability to move, and given that they express migration aspirations, the immobility of these 193 respondents can be related to a lack of the capability to move (this assumption of a lack of capability will be tested below). They can therefore be classified as “involuntary non-migrants/trapped populations.” The remaining 1263 non-migrants (87% of the non-migrants) did not express aspirations to move permanently in wave w2, and can therefore be considered “voluntary non-migrants” (if they have the capability to move) or “acquiescent non-migrants” (if they do not have the capability to move).

**Table 2:** Share of respondents classified as each of the four types of (im)mobility, relative to the total sample

N_total_ = 1515		**Migration capabilities**	
		Low	High
Migration aspirations	High	Involuntary immobility: *N* = 193 (13%)	Voluntary mobility: *N* = 59 (4%)
	Low	Acquiescent immobility: *N* = 76 (5%)	Voluntary immobility: *N* = 1187 (78%)

Empirically distinguishing between voluntary and acquiescent non-migrants is challenging because the “capability to move” is a latent construct that cannot be measured directly. To distinguish between the two groups, I asked those non-migrants who expressed a preference to stay in the village whether they would like to move away if they had sufficient resources. Seventy-six respondents indicated that they would prefer to move if they had sufficient resources, suggesting that these respondents (5% of non-migrants) can be classified as “acquiescent non-migrants” whose low migration aspirations can be related to a lack of migration capabilities. The remaining 82% of the non-migrants can be considered “voluntary non-migrants.” In a second, more indirect attempt to assess their “true” preference, I asked those non-migrants who themselves would prefer to stay in the village whether they would like their children to move away or stay in the village. Twenty percent said that they would like their children to move away. The main reasons given were a better standard of living and more earning opportunities elsewhere, education, and the threat of riverbank erosion (see Fig. S 4).

### Determinants of immobility being involuntary

**Fig. 4:**
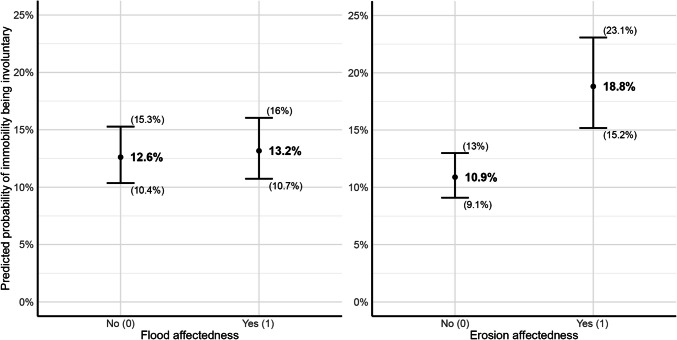
Predicted probabilities of immobility being involuntary, for flood affectedness (left panel, erosion affectedness held constant at mean value of 0.30) and erosion affectedness (right panel, flood affectedness held constant at mean value of 0.47), with 95% confidence intervals

Next, I investigate which factors influence the voluntariness of immobility and what characterizes the two^10^ groups of non-migrants. First, I model the influence of environmental shocks on whether immobility is voluntary/acquiescent or involuntary (model 1 in Table S 9). Respondents affected by erosion in the previous monsoon season are significantly more likely to become involuntary non-migrants (with voluntary/acquiescent non-migrants as the baseline category). The effect of flood affectedness remains insignificant. This could be explained by the fact that the impact of erosion is on average more severe than that of floods (see Fig. S 5). As the results of logistic regressions are difficult to interpret, Fig. 4 shows the predicted probabilities of respondents’ observed immobility being involuntary. While flood affectedness does not increase the likelihood of becoming involuntarily immobile, respondents who have been affected by erosion are on average 7.9 percentage points more likely to be involuntarily immobile than those who have not been affected by erosion.

Now, I test the assumption used above to identify involuntary non-migrants, namely that those who are non-migrants and express migration aspirations are immobile because they lack the capability to move. Model 2 in Table S 9 shows whether the two groups differ on three components of the capability to move. All three components are insignificant. Having tested the direct effects of environmental shocks and the capability to move, model 3 includes both constructs to test whether environmental shocks lead to involuntary immobility through a reduction of the capability to move (see the “Immobility in the face of environmental vulnerability” section). Indeed, the effect size of erosion affectedness is reduced compared to model 1, providing initial evidence that a reduction in socio-economic status may be the pathway through which environmental shocks lead to involuntary immobility. Finally, model 4 adds control variables and village-fixed effects to test the robustness of the effects. Erosion affectedness loses significance. This suggests that there are village-level processes that affect the relationship between environmental shocks and immobility. The only control variable that shows a significant influence is the migrant network, where respondents with a larger network are more likely to be involuntary non-migrants than those with a smaller network, supporting hypothesis H2b. Hypotheses H2a (place attachment) and H2c (risk aversion) cannot be confirmed, as both groups do not show a significant difference on these two variables.

### Mechanism behind immobility being involuntary

**Fig. 5:**
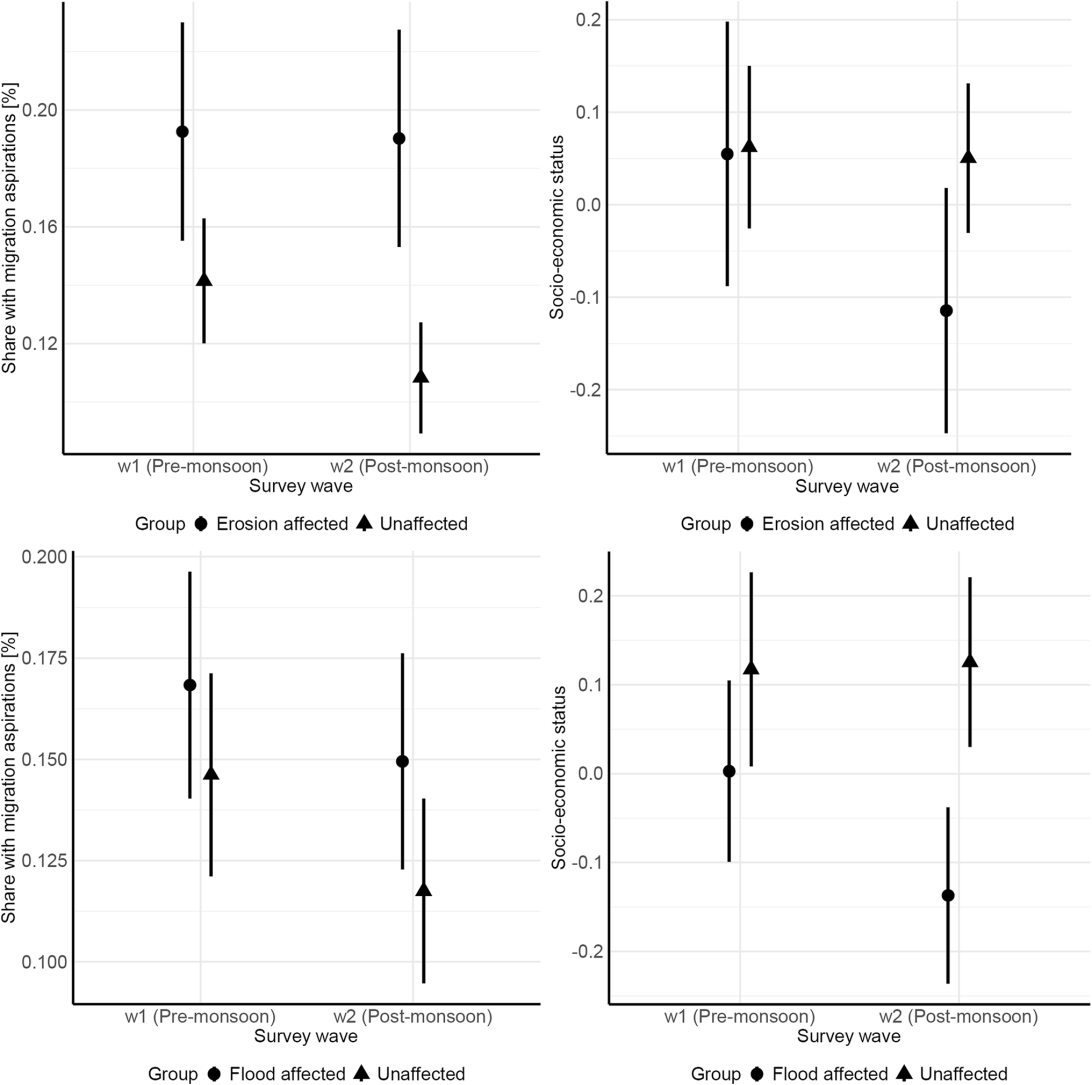
Difference-in-differences of migration aspirations (left column) and socio-economic status (right column), with treatment being erosion (upper row) and flood affectedness (lower row), respectively. Sample: non-migrants in wave w2. Whiskers indicate 95% confidence intervals

Extending the analysis of model 3 above, I explicitly test my core theoretical argument, namely that environmental shocks can lead to involuntary immobility by increasing migration aspirations while reducing the capability to move. Figure 5 illustrates, using a difference-in-differences approach, how migration aspirations and socio-economic status (as a proxy for the capability to move) change for those affected by the environmental shock (either erosion or flooding) compared to the unaffected control group. After the monsoon, those affected by erosion have, on average, unchanged levels of migration aspirations and (although not statistically significant) lower socio-economic status than before the monsoon, when compared to the control group (see model results in Table S 10). For flood affectedness, the effects are not significant (see model results in Table S 11). Overall, I hence find empirical support for my theoretical expectation that environmental shocks can lower socio-economic status, which in combination with existing migration aspirations can lead to trapped populations.

### Self-reported reasons for immobility

Finally, I compare the modelled determinants of immobility with respondents’ self-reported reasons for not moving. In the survey, respondents who said they had considered moving in the past but did not move were asked the open-ended question: “Why haven’t you gone?” According to the respondents’ replies, the most common reason for not moving despite having considered it is that erosion has not yet occurred (Fig. S 6). This shows, firstly, the strong link between erosion and aspirations and, secondly, that these respondents might have tried to move if erosion had occurred, depending on their capability to move.

The second most common self-reported reason for immobility is a lack of resources, either to pay for expenses in the migration location or to make the move itself. This underlines that the capability to move has a significant impact on the realization of migration aspirations. To better understand the resource constraints faced by those respondents who did not move due to a lack of resources, I asked them how much money they would need to realize their aspirations, and for what purpose. Eighty-six percent of the resource-constrained respondents would need less than 1 million Taka (~ $10,000), with a median of 500,000 Taka (~ $5000) (Fig. S 7). This is a significant amount, given the average monthly household income of around 8000 Taka (~ $80) reported in the survey. Over 80% of the respondents need money to buy/build a house or to buy land, respectively (Fig. S 8). More than 50% lack funds for relocation, while about 30% need funds to cover living expenses in the new location.

The third most cited group of reasons for staying in the village are land ownership as well as social and emotional ties with the village and its people (Fig. S 6).

## Discussion

Eighty-two percent of those who remain immobile after exposure to environmental change are classified as “voluntary non-migrants,” while 13% are classified as “involuntary non-migrants” based on their migration aspirations, and the remaining 5% are “acquiescent non-migrants.” This split is similar to other studies of immobility in environmentally exposed areas of Bangladesh, which find that 84% (Khatun et al. 2022) to 88% (Ahsan et al. 2022) of immobility is voluntary, based on respondents’ self-assessment. Erosion reduces the socio-economic status of affected households, hence potentially leading to trapped populations.

Respondents’ self-reported reasons for staying in the village rather than moving away reveal both instrumental (such as land ownership) and affective ties (such as love for family and fellow villagers), which may be related to an overall high level of place attachment. In addition, respondents cite resource constraints as a determinant of their mobility behavior, with both the availability of land and the resources required to purchase it limiting their freedom of choice. Similar to these findings, Mallick et al. (2022) conclude that land ownership, social ties, and economic strength are the strongest predictors of non-migration decisions in coastal Bangladesh. It thus appears that (im)mobility is driven by the interplay of social and economic capital.

Overall, this study provides evidence that the “capability to move” has a strong influence on immobility outcomes, supporting the notion that there may indeed be “trapped populations,” who would like to move away from exposed areas, but lack the means to do so. However, the relative proportion of “involuntary non-migrants” is much smaller than that of “voluntary non-migrants.” Given that the study was conducted in an area that is highly vulnerable to environmental change, with immediate and severe impacts on the livelihoods of the affected populations, this finding contradicts the notion that everyone who remains in such an exposed environment is “trapped.” Such simplistic narratives neglect the agency of populations and underestimate the strength of most people’s place attachment, which makes many prefer to stay rather than leave.

One challenge in studying immobility is the link between migration aspirations and capabilities. As discussed in the “Immobility in the face of environmental vulnerability” section, capabilities can influence aspirations. Similarly, aspirations can shift capabilities—for example, if someone who wants to move away invests in education to increase the chances of finding a job in the desired destination. These linkages blur the boundaries between the different forms of immobility—involuntary, voluntary, and acquiescent—both theoretically and empirically. In particular, if acquiescent non-migrants do not express migration aspirations due to a low capability to move, this raises the question of whether their immobility is truly voluntary or rather involuntary. In the latter case, the share of involuntary immobility found in this study would be underestimated. Empirically, this limits our ability to make a sharp distinction between voluntary and involuntary immobility. Theoretically, it invites us to think of voluntariness not as a binary tendency, but rather as a continuum. In a similar vein, Erdal and Oeppen (2018) discuss the difficulty of describing migration as either forced or voluntary, and suggest examining voluntariness as a continuum of experience.

A potential limitation of this study is that it focuses on individual-level data, neglecting the household and village dynamics of migration decision-making. This paper considers household heads as individuals and examines their migration aspirations. Since permanent migration usually involves the entire household, it seems plausible that the decision to migrate is discussed among the household members. For example, the household head may want to leave, but the spouse may prefer to stay, or vice versa.^11^ Similarly, the (non-)migration decisions of relatives, neighbors, or friends in the village may influence a household’s migration behavior. Initial evidence that household composition affects migration aspirations is provided by Rudolph et al. (2025) who find that respondents with more children have higher migration aspirations. However, their analysis does not capture the precise nature of migration decision-making within the household. Incorporating such broader dynamics into an analysis of (im)mobility will therefore be a worthwhile endeavor for future research.

There are two further limitations. First, the study looks at only one monsoon season and is therefore a snapshot of the short-term effects of environmental shocks. Over time, aspirations to move may either increase (e.g., if public services in the area decline) or decrease (e.g., if an embankment is built to prevent further erosion). In turn, people now classified as “voluntary/acquiescent” might become “involuntary non-migrants” and vice versa. Second, the analysis largely ignores political and institutional factors. For example, access to land is a strong determinant of people’s (im)mobility behavior. However, who is able to buy land or gain access to government-provided land is strongly influenced by local political processes, which should be disentangled in further research.

Finally, micro-level evidence for a specific case study raises the question of the extent to which the findings can be generalized to other settings. Undoubtedly, the Jamuna River is a very specific setting due to its high and unprecedented rates of erosion. Nevertheless, the process of irreversible erosion is similar to the phenomenon of coastal erosion associated with sea level rise. Moreover, flooding—the second major environmental event along the Jamuna—is a widespread phenomenon around the world. It therefore seems plausible that the findings of this study are relevant beyond the context of Bangladesh. However, as this is among the first papers to use individual-level migration aspirations to assess whether immobility is involuntary, this assumption remains to be confirmed by comparable studies for other geographical contexts and, importantly, other types of environmental and climatic changes.

## Conclusions

Placing this study in the wider context of a research agenda on climate mobilities, it emphasizes that we should not only consider “who can move and who cannot” (Cundill et al. 2021), but also who *wants* to move and who does not. In this sense, this study can be seen as a word of caution to researchers and policy makers not to prematurely and simplistically label certain populations as “trapped.” If the aim is to serve and support exposed populations in whatever they choose to do, it is essential to take into account their migration aspirations and the nuanced reasons for their immobility—only then can those who wish to stay be helped to adapt in situ, while those who prefer to migrate can be given the support they need to make their move a successful adaptation strategy. For example, direct assistance (e.g., cash transfers) could reduce economic barriers to migration, while planned relocation could provide a way out for people who want to leave but have no access to land or housing elsewhere. But whatever solution is proposed, it should be designed in close cooperation with the affected populations to ensure that their aspirations are adequately addressed.

## Supplementary Information

Below is the link to the electronic supplementary material.

Supplementary file1 (PDF 668 KB)

## Data Availability

The data and code underlying the analyses are published as Freihardt (2024b).

## Footnotes

1 Note that I prefer to use the term “(im)mobility” rather than “(non-)migration,” as the latter implies a binary choice between staying and leaving, which does not fully capture the multifaceted nature of mobility.

2 I base the following argumentation on an understanding of “environmental shocks” as environmental events which disrupt human or ecological systems and have a defined duration. While the core mechanisms (increase in aspirations, decrease in capabilities) might be similar for gradual changes such as sea-level rise or desertification, adaptation options might differ, potentially leading to a different theoretical link between gradual environmental changes and immobility.

3 Both sensitivity and adaptive capacity refer to the household’s capacities *before* the environmental shock occurs.

4 Note that “system” in these definitions corresponds to the household level in my case.

5 High response rate, random GIS sample of households within villages, and surveying of almost all inhabited survey locations with erosion risk along the eastern bank of the 250 km of the Jamuna River.

6 Amounts varied between survey waves, but were identical for all respondents within one particular wave.

7 Note that I have only considered aspirations to move *permanently*, i.e., I have filtered for those respondents who indicated in a follow-up question that they would like to leave the village with their entire family (as opposed to moving alone for a temporary work opportunity while the family remains in the village).

8 Given the recurrent nature of floods and erosion along the Jamuna River, they may not appear to be “shocks” that take people by surprise. However, they can have drastic impacts such as a loss of house and/or land, which is a significant “shock” for people’s livelihoods.

9 For an assessment of how the populations along the Jamuna perceive erosion, see Freihardt (2024a).

10 For simplicity, I combine the two groups without migration aspirations (voluntary/acquiescent non-migrants) and compare them with those with aspirations (involuntary non-migrants).

11 We might assume that the household head communicates the migration aspiration of the entire household. However, this assumption remains to be verified.
