# Supplementary material for: Trapped by climate change? (In)voluntary immobility in Bangladesh
Source: Reg Environ Change. 2025 Sep 8;25(4):117. doi: 10.1007/s10113-025-02452-3 (PMC12417244; doi:10.1007/s10113-025-02452-3)
Supplement: Supplementary file 1 — Supplementary file1 (PDF 668 KB) [file 10113_2025_2452_MOESM1_ESM.pdf]

# Supplementary Information for

# Trapped by climate change? (In)voluntary immobility in Bangladesh

Jan Freihardt[1]

[1]ETH Zurich, Zurich, Switzerland

*Correspondence to*: Jan Freihardt, ETH Zurich, Center for Comparative and International Studies (CIS), Haldeneggsteig 4, 8092 Zurich, Switzerland, fjan@ethz.ch

Published in: Regional Environmental Change (2025), https://doi.org/10.1007/s10113-025-02452-3

# Appendix A: Selection of study locations

I selected participants in a multi-stage cluster design. In the first stage, I selected 36 locations along the easternmost riverbank line of the Jamuna because the rates of riverbank erosion are higher along the eastern than along the western riverbank due to differences in floodplain materials (CEGIS, 2018; Sarker et al., 2014). I identified this line using the most recent satellite imagery available. Villages on chars (sandy islands in the river) were not considered since char populations have adapted their livelihoods to the yearly recurring flood and erosion events (Alam et al., 2017; Islam et al., 2015).

Along this line, I defined 250 sampling points with a one-kilometer distance along the whole stretch of the river (from the border with India in the north to the convergence of Ganges and Jamuna in the south). Ideally, I would have drawn the 36 study locations randomly from this pool of 250 stretches. However, a visual, satellite-based analysis revealed that not all of these 250 stretches were suitable for my study purpose. Therefore, I evaluated each of the 250 stretches with respect to the following two criteria: First, survey feasibility, that is, whether there were enough settlements (= at least 75 houses) in the 200 m stretch inland; and second, the ex-ante risk for riverbank erosion, in particular, whether there was a clear indication of a permanent embankment structure that prevents erosion and whether there was a char/large sandbank in front of the stretch that blocks erosion.

Stretches for which the satellite analysis showed that at least one of these criteria was violated were excluded from the pool. This reduced the pool size from 250 to 79 stretches. For some of these 79 stretches, not all criteria could be clearly evaluated from the satellite images due to insufficient image resolution. Therefore, the final screening was done on the ground during a field visit. Six stretches could not be visited due to their remote location. Of the remaining 73 stretches, 29 were excluded after the field visit due to a violation of at least one of the three criteria. One stretch was used for training the enumerators, leaving 43 stretches suitable for my sample. Due to time constraints during the fieldwork, not all 43 stretches could be included in the sample. Therefore, I chose 36 stretches such that they were well distributed along the entire length of the Jamuna. An overview of the 36 locations is provided in Fig. 1A. Table S 1 provides a list of all 79 stretches initially in the sample, including whether they were part of the final sample and – if not – the reason for their exclusion.

At each of the 36 locations, households were sampled using a stratified random spatial sampling design to survey households located within three zones defined by distance from the shoreline. This design allowed capturing potential effects of different ex-ante erosion risk levels on perceptions of environmental changes. At each location, the three zones were constructed by shifting the shoreline inland by 50 m, 100 m and 200 m, respectively. Consequently, each sampling zone has an extent of 1 km in the direction of flow and of 200 m inland.

Within each of the three zones, a spatially explicit sample was generated following the procedure outlined by Crawford et al. (2020). Specifically, a set of 25 random latitude-longitude points per zone was created using ArcMap software (with a minimum distance of 10 m between points). In the field, enumerators navigated to these points using smartphones. Having arrived at the point, they selected the house closest to that point based on visual estimation. This household was subsequently interviewed (see Fig. S 1 for the distribution of the households' distance from the riverbank). If a household declined participation or if the household head was not available at two contact attempts, the enumerator continued to the next closest household, in reference to the starting point. Within each household, the household head was interviewed, defined as the decision-maker within the household.

Table S 1: List of 79 stretches initially in the sample.

| Site | Latitude | Longitude | District | Sampled | Reason for exclusion |
|---|---|---|---|---|---|
| 1 | 23.84858386 | 89.77728293 | Manikganj | Yes | |
| 2 | 23.85741259 | 89.7761692 | Manikganj | | Suitable, excluded for time constraints |
| 3 | 23.8659592 | 89.77336891 | Manikganj | Yes | |
| 4 | 23.8741437 | 89.7696434 | Manikganj | | Training site |
| 5 | 23.88306306 | 89.76938992 | Manikganj | | Suitable, excluded for time constraints |
| 6 | 23.89188075 | 89.77113454 | Manikganj | | Embankment |
| 7 | 23.93977265 | 89.77300493 | Manikganj | | Not visited |
| 8 | 23.96265272 | 89.75980911 | Manikganj | | Char/sandbank |
| 9 | 23.971464 | 89.75801152 | Manikganj | | Char/sandbank |
| 10 | 23.97946232 | 89.76071457 | Manikganj | Yes | |
| 11 | 23.98646574 | 89.76636763 | Manikganj | | Not enough settlement |
| 12 | 23.99336663 | 89.77212095 | Manikganj | | Not enough settlement |
| 13 | 24.00092974 | 89.77700303 | Manikganj | | Not enough settlement |
| 14 | 24.0080967 | 89.78240531 | Manikganj | | Not enough settlement |
| 15 | 24.01468743 | 89.78848452 | Tangail | Yes | |
| 16 | 24.01998107 | 89.79575917 | Tangail | | Not visited |
| 17 | 24.02782786 | 89.79999907 | Tangail | | Not visited |
| 18 | 24.03669272 | 89.80143637 | Tangail | Yes | |
| 19 | 24.04554666 | 89.80018357 | Tangail | Yes | |
| 20 | 24.05359151 | 89.79620979 | Sirajganj | Yes | |
| 21 | 24.06191619 | 89.79537304 | Sirajganj | | Not enough settlement; char/sandbank |
| 22 | 24.07061319 | 89.79705522 | Sirajganj | Yes | |
| 23 | 24.07936755 | 89.79571167 | Sirajganj | Yes | |
| 24 | 24.08826802 | 89.79443957 | Sirajganj | | Not enough settlement; char/sandbank |
| 25 | 24.09651555 | 89.79100124 | Sirajganj | | Char/sandbank |
| 26 | 24.10416064 | 89.78625988 | Sirajganj | | Char/sandbank |
| 27 | 24.11252358 | 89.78760715 | Sirajganj | | Not enough settlement; embankment |
| 28 | 24.1199595 | 89.79245961 | Sirajganj | | Embankment |
| 29 | 24.12686204 | 89.79824051 | Sirajganj | | Embankment |
| 30 | 24.13388967 | 89.80382386 | Sirajganj | | Embankment |
| 31 | 24.14197229 | 89.80777083 | Sirajganj | | Embankment |
| 32 | 24.15055663 | 89.81028585 | Tangail | | Not enough settlement; embankment |
| 33 | 24.15953767 | 89.81062441 | Tangail | | Suitable, excluded for time constraints |
| 34 | 24.16836954 | 89.80947906 | Tangail | Yes | |
| 35 | 24.17727796 | 89.80949719 | Tangail | Yes | |
| 36 | 24.2295696 | 89.78659759 | Tangail | | Not visited |
| 37 | 24.23854823 | 89.7860587 | Tangail | | Not visited |
| 38 | 24.34224707 | 89.81162957 | Tangail | Yes | |
| 39 | 24.35117757 | 89.81254109 | Tangail | Yes | |
| 40 | 24.36017152 | 89.81221315 | Tangail | Yes | |
| 41 | 24.36889043 | 89.81040512 | Tangail | | Not enough settlement; embankment |
| 42 | 24.38032687 | 89.8048785 | Tangail | | Embankment |
| 43 | 24.43313402 | 89.8199736 | Tangail | | Char/sandbank |
| 44 | 24.44195819 | 89.82141819 | Tangail | Yes | |

| | | | | | |
|---|---|---|---|---|---|
| 45 | 24.45047689 | 89.82397444 | Tangail | Yes | |
| 46 | 24.49471362 | 89.84519411 | Tangail | Yes | |
| 47 | 24.50358737 | 89.84590586 | Tangail | Yes | |
| 48 | 24.51181581 | 89.84245001 | Tangail | | Embankment |
| 49 | 24.58706697 | 89.81975046 | Jamalpur | | Not enough settlement; embankment |
| 50 | 24.59501471 | 89.81563181 | Jamalpur | | Not enough settlement; embankment |
| 51 | 24.60315021 | 89.81184187 | Jamalpur | | Embankment |
| 52 | 24.61077011 | 89.80728877 | Jamalpur | | Embankment |
| 53 | 24.61961004 | 89.80620216 | Jamalpur | | Embankment |
| 54 | 24.9010855 | 89.65554932 | Bogra | | Not visited |
| 55 | 24.96551006 | 89.66537782 | Bogra | Yes | |
| 56 | 24.97114163 | 89.67177488 | Bogra | Yes | |
| 57 | 24.9745598 | 89.68009865 | Jamalpur | | Not enough settlement |
| 58 | 24.98684177 | 89.69272319 | Jamalpur | | Not enough settlement |
| 59 | 25.00071823 | 89.70405209 | Jamalpur | | Char/sandbank |
| 60 | 25.21211443 | 89.72276084 | Jamalpur | | Not enough settlement |
| 61 | 25.22079034 | 89.7204164 | Jamalpur | Yes | |
| 62 | 25.22963506 | 89.7196273 | Jamalpur | Yes | |
| 63 | 25.33554382 | 89.72866088 | Gaibandha | Yes | |
| 64 | 25.36243599 | 89.7450544 | Jamalpur | Yes | |
| 65 | 25.38816632 | 89.74946849 | Kurigram | | Suitable, excluded for time constraints |
| 66 | 25.39617635 | 89.75357157 | Kurigram | Yes | |
| 67 | 25.40397791 | 89.75805218 | Kurigram | Yes | |
| 68 | 25.41161802 | 89.76276468 | Kurigram | Yes | |
| 69 | 25.42006097 | 89.76266573 | Kurigram | Yes | |
| 70 | 25.58524338 | 89.80014675 | Kurigram | Yes | |
| 71 | 25.59256635 | 89.80534785 | Kurigram | Yes | |
| 72 | 25.60067086 | 89.80889396 | Kurigram | Yes | |
| 73 | 25.60950232 | 89.80727078 | Kurigram | Yes | |
| 74 | 25.62799682 | 89.78911681 | Kurigram | Yes | |
| 75 | 25.63403951 | 89.78356735 | Kurigram | Yes | |
| 76 | 25.64292418 | 89.78384213 | Kurigram | Yes | |
| 77 | 25.65177817 | 89.78241922 | Kurigram | Yes | |
| 78 | 25.66053126 | 89.78367557 | Kurigram | Yes | |
| 79 | 25.66861671 | 89.78750184 | Kurigram | Yes | |

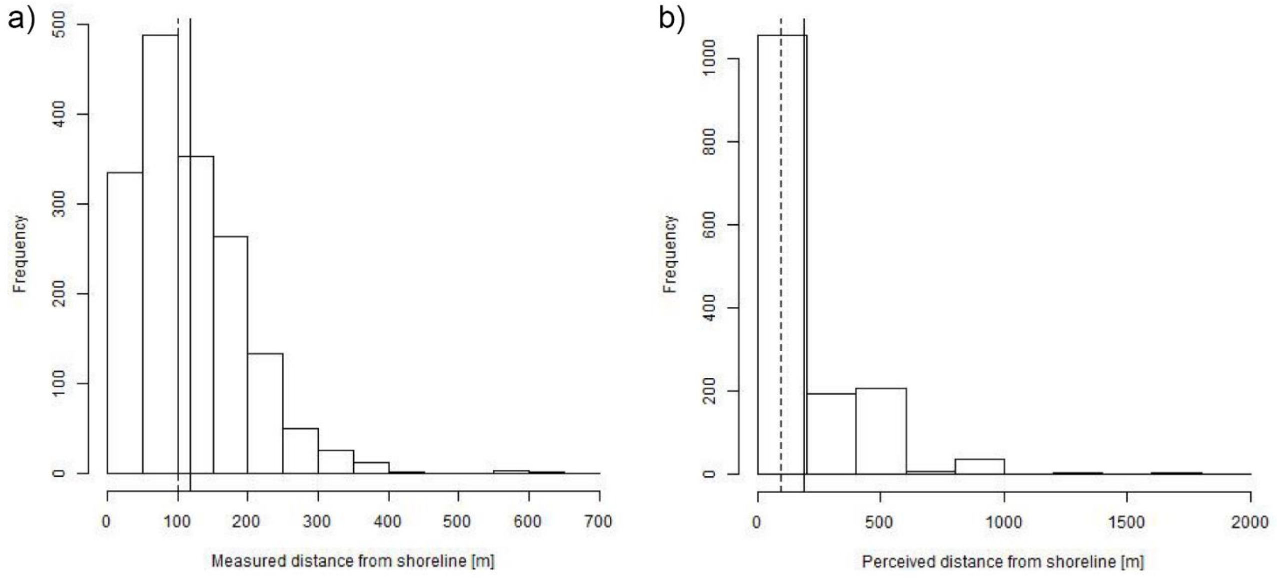


Fig. S 1: a) Measured and b) perceived distance of households from next closest riverbank of Jamuna River. Vertical lines: median (dashed) and mean (solid).

# Appendix B: Comparison of objective erosion data to self-reported impacts

A challenge for studying the link between environmental events and (im)mobility is that there exist no objective data on exposure. Satellite imagery on flood extent and depth is not available at the household scale, as cloud cover during the monsoon prevents such data from being gathered. Additionally, the Bangladesh government or other organizations do not provide flood maps with the detail required. Satellite imagery on erosion occurrence can be gathered by comparing riverbeds before and after the monsoon (Freihardt & Frey, 2023). However, in both cases, even if we had perfect exposure data on erosion and flood occurrence available, this would not tell us to what extent individual households are affected. For example, flood or erosion of uninhabited land will directly affect households that farm on or own this land – but such objective data cannot be linked to individuals given that individual-specific land-use maps are not available. Hence, I primarily rely on self-reported exposure directly inquired from respondents, which gives a nuanced picture on whether respondents report to have been affected at all, and to what extent. As I rely on straightforward factual questions ("Were you affected by erosion/floods?" and "What was the first, second and third most important impact on your household?"), and as the communicated scientific study goals provide no direct incentives for respondents to under- or overstate affectedness (other than with NGO- or government-sponsored surveys), I am confident that this approach leads to a sincere measure of affectedness. Still, I acknowledge that such self-reported assessments can exhibit biases. For example, it would be particularly worrisome if households that have migrated self-justify the move with flood or erosion impact, and subsequently overstate such impacts.

Therefore, I draw on one objective, and likely accurate measure of an erosion impact, namely the loss of house, to verify whether a self-reported loss of house and a satellite-based indication that the GIS location of the respondent's home was eroded coincide.

Specifically, I use respondents' house coordinates as registered during wave 1 and the satellite-based erosion assessment tool developed by Freihardt and Frey (2023) to identify those respondents whose house was eroded during the 2021 monsoon. First, I compare this objective indicator of house loss to whether respondents self-reported any erosion impacts (Table S 2). Out of the 158 respondents who objectively lost their house, 144 (91%) self-reported to have been affected by erosion. The remaining 9% did not report impacts despite the

satellite imagery identifying them as having lost their house. Second, I compare the objective data to the more specific impact categories (Table S 3). Out of the 158 respondents who objectively lost their house, 109 (69%) self-reported to have lost their house. Out of 1,446 respondents whose house location did not get eroded according to the satellite imagery, 1,406 (97%) did not self-report a loss of house. Overall, this means that self-reported and objective data are coherent for 94% of all respondents. Forty-nine respondents (3% of all respondents) indicated no loss of house, despite the satellite analysis revealing that their house location has been eroded. However, considering which specific impacts were indicated by this subset of respondents (Figure A.2), the majority of them still indicated a severe or medium impact (permanent displacement, total loss of land, damage of the house) – all of which are plausible impacts for the case where their house location has been eroded. The remaining 40 respondents (3% of all respondents) indicated a loss of their house which was not confirmed by the satellite analysis. Several reasons might explain this mismatch: First, respondents might have lost their house in an earlier monsoon season and mistakenly indicated it as an impact of the 2021 monsoon. Second, technical problems (e.g., recording the coordinates or extracting the bank line from satellite imagery) might result in mis-classifications of the satellite analysis. Third, enumerators might have mistakenly clicked the wrong category. Overall, however, these analyses increase my confidence in relying the main analyses on self-reported impacts, given that only 3% of all respondents can be classified as not in line with objective data.

Table S 2: Cross-tabulation of any self-reported erosion impacts and objectively determined house loss.

| | **Objectively lost their house** | | |
|---|---|---|---|
| **Self-reported any erosion impact** | No | Yes | Row Total |
| No | 1,064 (66.3%) | 14 (0.9%) | 1,078 |
| Yes | 382 (23.8%) | 144 (9.0%) | 526 |
| Column Total | 1,446 | 158 | 1,604 |

Table S 3: Cross-tabulation of self-reported erosion-induced house loss and objectively determined house loss.

| | **Objectively lost their house** | | |
|---|---|---|---|
| **Self-reported loss of house** | No | Yes | Row Total |
| No | 1,406 (87.7%) | 49 (3.1%) | 1,455 |
| Yes | 40 (2.5%) | 109 (6.8%) | 149 |
| Column Total | 1,446 | 158 | 1,604 |

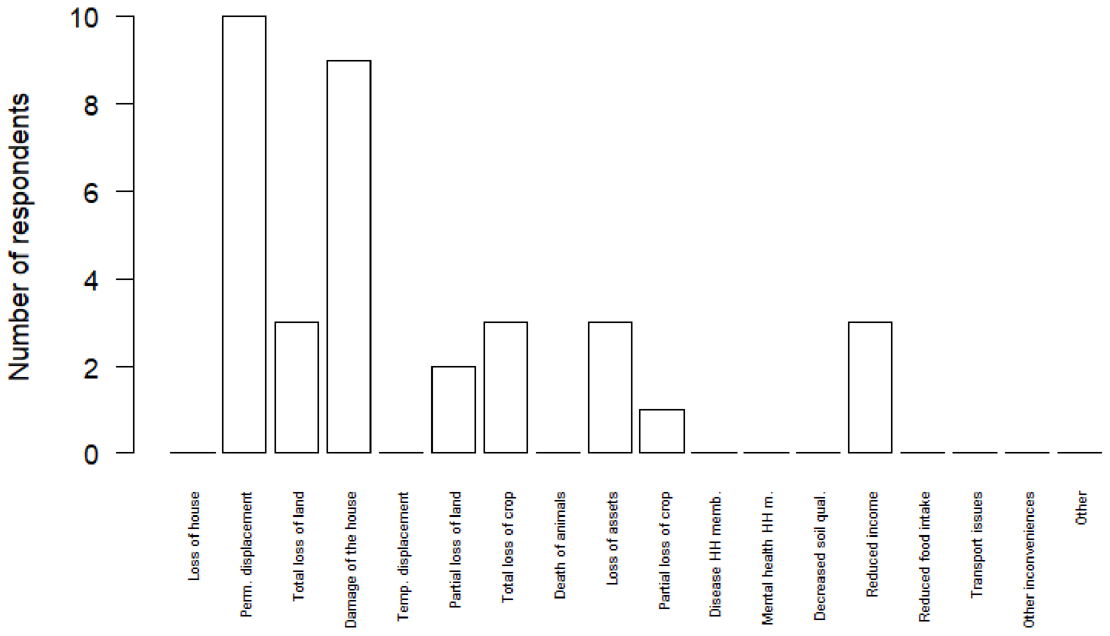


Fig. S 2: Primary erosion impact as self-reported by those respondents who did not indicate to have lost their house, but for whom the satellite-based analysis revealed a house loss.

# Appendix C: Supporting figures and tables

Table S 4: Overview of interview status of respondents in wave w2 – both for the overall sample and split by migration status.

| | | **Overall** | **…of which in wave w2** | | | | | |
|---|---|---|---|---|---|---|---|---|
| | | | re-interviewed | currently unavailable | refused to be interviewed | temporary migrants | not found | died |
| **Total sample** | Number | 1688 | 1515 | 37 | 12 | 75 | 45 | 4 |
| | Share [%] | 100 | 89.8 | 2.2 | 0.7 | 4.4 | 2.7 | 0.2 |
| **Non-migrants** | Number | 1524 | 1456 | 13 | 9 | 0 | 42 | 4 |
| | Share [%] | 90.3 | 95.5 | 0.9 | 0.6 | 0.0 | 2.8 | 0.3 |
| **Migrants** | Number | 164 | 59 | 24 | 3 | 75 | 3 | 0 |
| | Share [%] | 9.7 | 36.0 | 14.6 | 1.8 | 45.7 | 1.8 | 0.0 |

Table S 5: Summary statistics of relevant variables.

| **Variable** | **N** | **Mean** | **Std. Dev.** | **Min** | **Pctl. 25** | **Pctl. 75** | **Max** |
|---|---|---|---|---|---|---|---|
| Permanent move between w1 and w2? | 1,515 | 0.039 | 0.19 | 0 | 0 | 0 | 1 |
| Personal affectedness by erosion 2021 (w2) | 1,515 | 0.32 | 0.47 | 0 | 0 | 1 | 1 |
| Personal affectedness by flood 2021 (w2) | 1,514 | 0.48 | 0.5 | 0 | 0 | 1 | 1 |
| Migration aspirations (w1) | 1,515 | 0.16 | 0.37 | 0 | 0 | 0 | 1 |
| Migration aspirations (w2) | 1,508 | 0.13 | 0.33 | 0 | 0 | 0 | 1 |
| Socio-economic status (w1) | 1,288 | 0.05 | 1.3 | -3.3 | -0.81 | 0.71 | 9.5 |
| Socio-economic status (w2) | 1,285 | 0.00047 | 1.2 | -2.7 | -0.58 | 0.58 | 10 |
| Sex | 1,515 | 0.86 | 0.34 | 0 | 1 | 1 | 1 |
| Marital status | 1,515 | 0.91 | 0.28 | 0 | 1 | 1 | 1 |
| Age | 1,512 | 48 | 14 | 18 | 38 | 57 | 95 |
| Education level | 1,514 | 0.86 | 1.3 | 0 | 0 | 1 | 5 |
| Income env.-dependent? (w1) | 1,377 | 0.59 | 0.49 | 0 | 0 | 1 | 1 |
| Place attachment (w1) | 1,514 | 4.4 | 0.73 | 1 | 4 | 5 | 5 |
| Risk preference (w1) | 1,496 | 3.4 | 1.3 | 1 | 2 | 4 | 5 |
| Migrant network (w2) | 1,498 | 2.6 | 1.1 | 1 | 2 | 3 | 5 |

Table S 6: Dimensions and components of principal component analysis used to assess respondents' socio-economic status.

| **Dimension** | **Components** |
|---|---|
| Valuables | 1.5*number of mobile phones + radio + 2*TV + 3*laptop + fan + battery + 2*solar panel + lighbulb + watch |
| Lifestock units (LSU) | 1*number of cows + 0.1*number of goats + 0.014* number of hens + 0.005*number of pigeons |
| Transport equipment | bicycle + 1.5*rickshaw + 1.5*van + 2*autobike + 2*motorbike + 3*CNG |
| Productive equipment | tractor + fishing net + boat |
| Total land size [ha] | - |
| Building quality | quality of roof (1=hay / tin; 1.5=wood / plastic; 2=concrete / steel; 3=tiles) + |
| | quality of walls (1=jute / mud / bamboo; 1.5=wood; 2=concrete / iron; 3=brick) |
| Housing quality | house owned + 0.5*latrine + 0.5*connection to electricity grid |

Table S 7: Impact types used to construct four categories of impact severity.

| **Impact severity** | **Impact type** |
|---|---|
| Strong impact | - Loss of house<br>- Permanent displacement<br>- Total loss of land |
| Medium impact | - Damage to house<br>- Temporal displacement<br>- Partial loss of land<br>- Total loss of crop<br>- Death/disease of animals<br>- Loss of assets |
| Low impact | - Partial loss of crop<br>- Decrease of soil quality<br>- Difficulties with transport<br>- Disease/injury of household members<br>- Reduced income<br>- Reduced food intake<br>- Mental health impacts<br>- Other impact/inconvenience |
| No impact | - No impact reported |

Table S 8: Correlation matrix of main variables. Correlation coefficients larger than 0.3 / smaller than -0.3 are highlighted.

| | Age | Sex | Education | Marital status | Income env. dep.? | Place attachment | Risk preference | Socio-economic status | Migration aspirations | Migrant network |
|---|---|---|---|---|---|---|---|---|---|---|
| Age | 1.00 | 0.13 | -0.14 | -0.05 | 0.07 | -0.02 | -0.08 | -0.01 | 0.02 | 0.09 |
| Sex | 0.13 | 1.00 | 0.01 | 0.62 | 0.16 | 0.10 | 0.00 | 0.16 | 0.05 | 0.00 |
| Education | -0.14 | 0.01 | 1.00 | 0.02 | -0.28 | 0.08 | 0.04 | 0.27 | 0.05 | 0.02 |
| Marital status | -0.05 | 0.62 | 0.02 | 1.00 | 0.05 | 0.07 | 0.03 | 0.16 | 0.08 | -0.01 |
| Income env. dep.? | 0.07 | 0.16 | -0.28 | 0.05 | 1.00 | -0.02 | 0.00 | -0.08 | -0.08 | 0.01 |
| Place attachment | -0.02 | 0.10 | 0.08 | 0.07 | -0.02 | 1.00 | 0.10 | 0.15 | -0.04 | -0.03 |
| Risk preference | -0.08 | 0.00 | 0.04 | 0.03 | 0.00 | 0.10 | 1.00 | 0.10 | 0.00 | 0.00 |
| Socio-economic status | -0.01 | 0.16 | 0.27 | 0.16 | -0.08 | 0.15 | 0.10 | 1.00 | -0.06 | 0.03 |
| Migration aspirations | 0.02 | 0.05 | 0.05 | 0.08 | -0.08 | -0.04 | 0.00 | -0.06 | 1.00 | -0.03 |
| Migrant network | 0.09 | 0.00 | 0.02 | -0.01 | 0.01 | -0.03 | 0.00 | 0.03 | -0.03 | 1.00 |

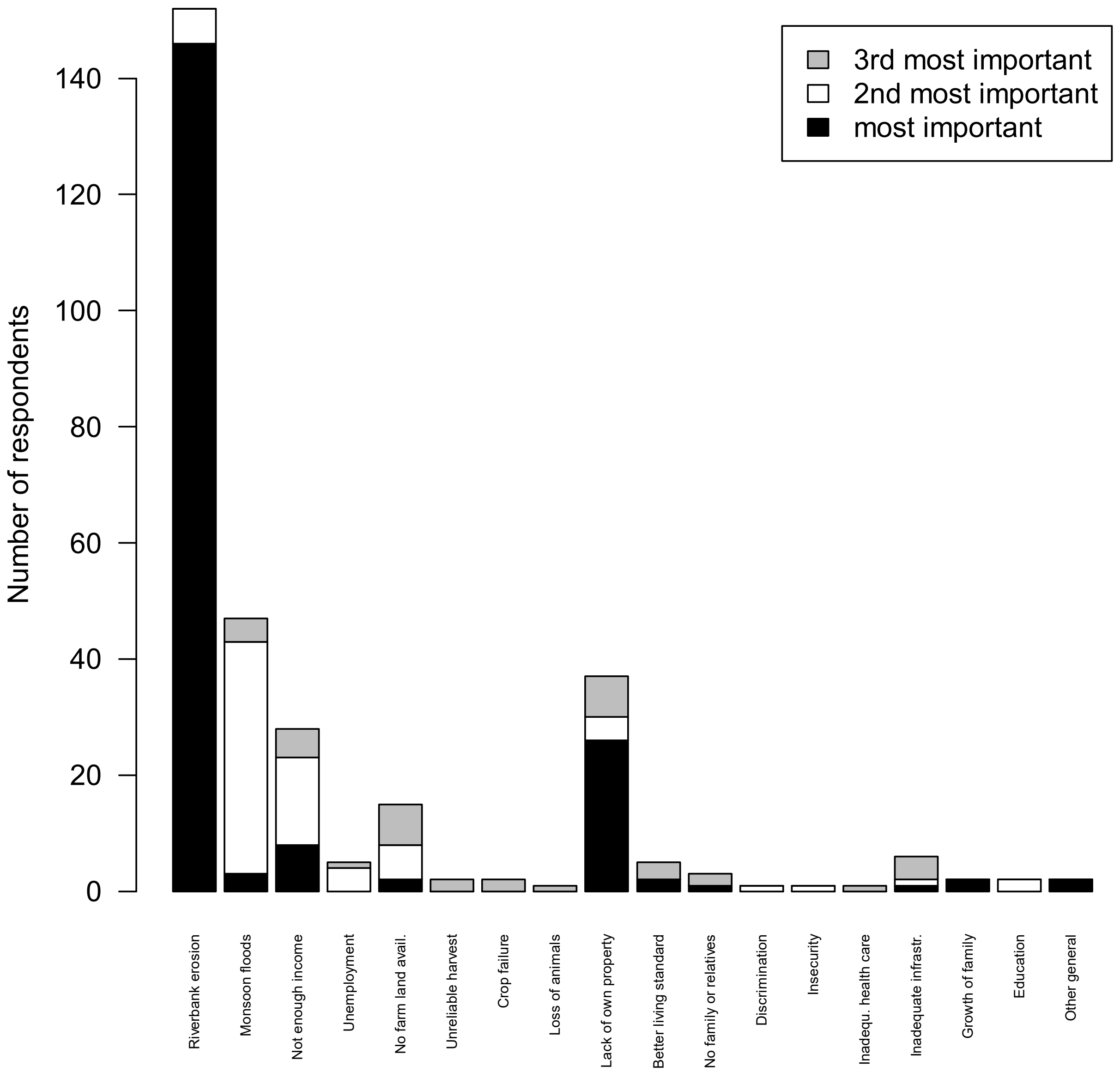


Fig. S 3: Reasons for wanting to leave stated by those respondents who indicated in wave w2 that they would like to permanently leave the village.

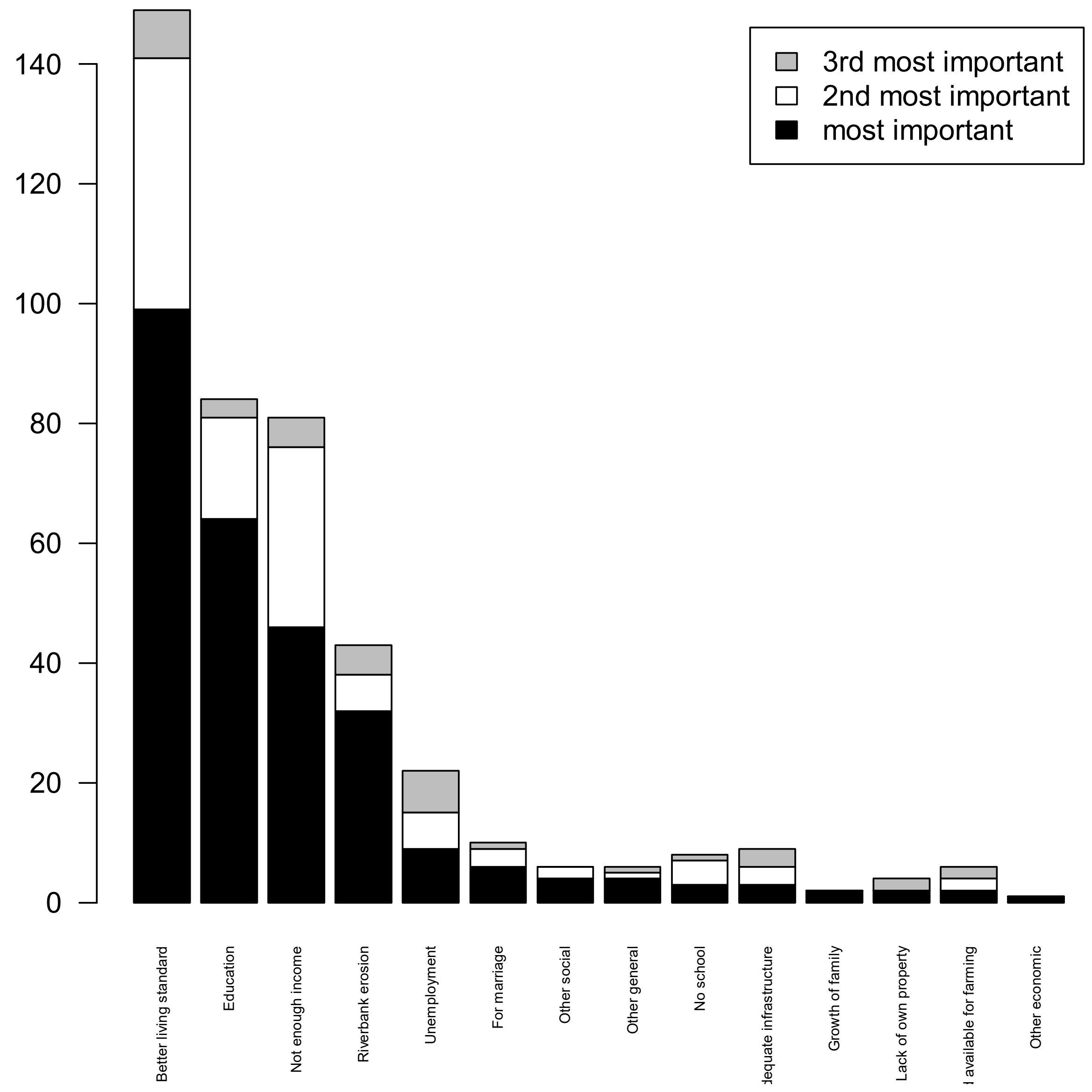


Fig. S 4: Reasons stated by respondents why they would like their children to move away despite themselves indicating no migration aspirations.

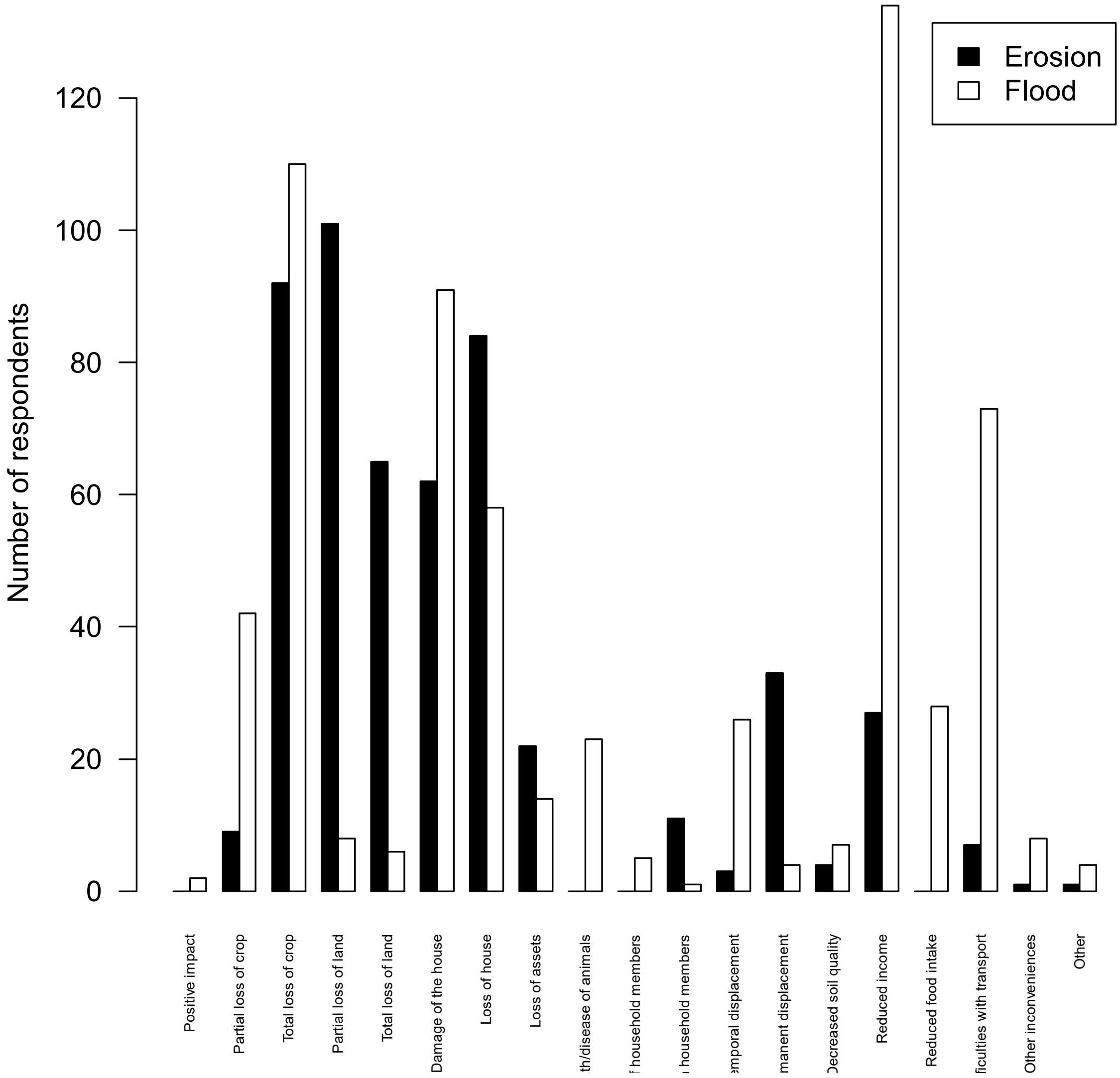


Fig. S 5: Most important impacts of the 2021 erosion and flood, respectively, as self-reported by respondents.

Table S 9: Determinants of whether non-migration is voluntary or involuntary as classified according to non-migrants' migration aspirations (logistic regression models; full model results). Model 1: including environmental shocks. Model 2: including capability to move. Model 3: including environmental shocks and capability to move. Model 4: including environmental shocks, capability to move, controls and village-fixed effects.

| | *Dependent variable:* | | | |
|---|---|---|---|---|
| | Immobility involuntary? (n/y) | | | |
| | (1) | (2) | (3) | (4) |
| Erosion impact (n/y) | $0.64^{***}$ | | $0.59^{***}$ | -0.06 |
| | (0.19) | | (0.18) | (0.25) |
| Flood impact (n/y) | 0.05 | | 0.07 | -0.003 |
| | (0.15) | | (0.18) | (0.22) |
| Socio-econ. status | | -0.09 | -0.09 | -0.05 |
| | | (0.07) | (0.07) | (0.08) |
| Sex (f/m) | | 0.15 | 0.16 | -0.20 |
| | | (0.25) | (0.26) | (0.39) |
| Educ: primary | | -0.27 | -0.22 | -0.26 |
| | | (0.25) | (0.25) | (0.30) |
| Educ: secondary | | 0.08 | 0.10 | 0.19 |
| | | (0.26) | (0.26) | (0.32) |
| Educ: SSC passed | | -0.29 | -0.25 | -0.85 |
| | | (0.49) | (0.49) | (0.71) |
| Educ: HSC passed | | -0.49 | -0.51 | -0.25 |
| | | (0.54) | (0.54) | (0.62) |
| Educ: university | | 0.13 | 0.18 | 0.21 |
| | | (0.46) | (0.47) | (0.53) |
| Married? (n/y) | | | | 0.61 |
| | | | | (0.48) |
| Age: 31-40 yr | | | | -0.08 |
| | | | | (0.38) |
| Age: 41-50 yr | | | | 0.17 |
| | | | | (0.38) |
| Age: 51-60 yr | | | | 0.10 |

| | |
|---|---|
| | (0.40) |
| Age: 61+ yr | 0.16 |
| | (0.40) |
| Income env.-dep.? (n/y) | -0.05 |
| | (0.22) |
| Attachment (1-5) | -0.07 |
| | (0.14) |
| Risk pref. (1-5) | -0.03 |
| | (0.08) |
| Migrant network (1-5) | 0.23** |
| | (0.09) |
| Village 5 | -15.14 |
| | (947.50) |
| Village 6 | -0.49 |
| | (0.92) |
| Village 7 | -0.63 |
| | (0.82) |
| Village 8 | 2.93*** |
| | (0.71) |
| Village 9 | 0.30 |
| | (0.75) |
| Village 10 | 0.17 |
| | (0.96) |
| Village 11 | 1.57** |
| | (0.65) |
| Village 12 | 0.57 |
| | (0.76) |
| Village 13 | -0.58 |
| | (0.92) |
| Village 14 | -15.52 |
| | (752.49) |
| Village 15 | 1.27 |
| | (0.85) |
| Village 16 | -0.85 |
| | (1.18) |

| | |
|---|---|
| Village 17 | 2.30* |
| | (1.20) |
| Village 18 | 2.54* |
| | (1.38) |
| Village 19 | -0.61 |
| | (0.83) |
| Village 20 | 0.32 |
| | (0.70) |
| Village 21 | 2.02** |
| | (0.99) |
| Village 22 | 0.31 |
| | (0.67) |
| Village 23 | 0.29 |
| | (0.69) |
| Village 24 | -0.57 |
| | (0.76) |
| Village 25 | -0.21 |
| | (0.83) |
| Village 26 | 0.13 |
| | (0.75) |
| Village 27 | -0.25 |
| | (0.93) |
| Village 28 | 0.10 |
| | (0.71) |
| Village 29 | 0.62 |
| | (0.75) |
| Village 30 | -0.60 |
| | (0.82) |
| Village 31 | -0.39 |
| | (0.73) |
| Village 32 | -0.71 |
| | (0.77) |
| Village 33 | -0.91 |
| | (0.92) |
| Village 34 | -0.91 |
| | (0.92) |
| Village 35 | 0.49 |
| | (0.88) |

| | | | | |
|---|---|---|---|---|
| Village 36 | | | | 0.79 |
| | | | | (0.69) |
| Intercept | -2.12*** | -1.93*** | -2.19*** | -2.63*** |
| | (0.14) | (0.25) | (0.27) | (1.00) |
| Village FE? | No | No | No | Yes |
| Observations | 1,455 | 1,231 | 1,230 | 1,097 |
| Log Likelihood | -561.07 | -487.97 | -481.43 | -374.65 |
| Akaike Inf. Crit. | 1,128.14 | 991.93 | 982.85 | 851.29 |

*Note:* *p<0.1; **p<0.05; ***p<0.01
Standard errors clustered by village. Baseline age: 18-30 yr. Baseline education: no education. Baseline village: village 4. (n/y) – (no/yes), (f/m) – (female/male). Sample: non-migrants in wave 2.

Table S 10: Difference-in-differences of migration aspirations (model 1) and socio-economic status (model 2), with treatment being **erosion affectedness**. Sample: non-migrants in wave w2.

| | *Dependent variable:* | |
|---|---|---|
| | asp | SES |
| | (1) | (2) |
| Erosion affectedness | 0.05** | -0.01 |
| | (0.02) | (0.08) |
| Time | -0.03** | -0.01 |
| | (0.02) | (0.06) |
| Erosion * time | 0.03 | -0.16 |
| | (0.03) | (0.11) |
| Constant | 0.14*** | 0.06 |
| | (0.01) | (0.04) |
| Observations | 2,912 | 2,517 |
| $R^2$ | 0.01 | 0.002 |
| Adjusted $R^2$ | 0.01 | 0.001 |
| Residual Std. Error | 0.35 (df = 2908) | 1.25 (df = 2513) |
| F Statistic | 8.83*** (df = 3; 2908) | 1.98 (df = 3; 2513) |
| *Note:* | | *p<0.1; **p<0.05; ***p<0.01 |

Table S 11: Difference-in-differences of migration aspirations (model 1) and socio-economic status (model 2), with treatment being **flood affectedness**. Sample: non-migrants in wave w2.

| | *Dependent variable:* | |
|---|---|---|
| | asp | SES |
| | (1) | (2) |
| Flood affectedness | 0.02 | -0.11 |
| | (0.05) | (0.07) |
| Time | -0.03 | -0.03 |
| | (0.03) | (0.07) |
| Flood * time | 0.01 | -0.07 |
| | (0.04) | (0.10) |
| Constant | $0.15^{***}$ | $0.12^{**}$ |
| | (0.01) | (0.05) |
| Observations | 2,910 | 2,516 |
| $R^2$ | 0.003 | 0.004 |
| Adjusted $R^2$ | 0.002 | 0.003 |
| Residual Std. Error | 0.35 (df = 2906) | 1.25 (df = 2512) |
| F Statistic | $2.62^{**}$ (df = 3; 2906) | $3.70^{**}$ (df = 3; 2512) |
| *Note:* | | $^{*}$p<0.1; $^{**}$p<0.05; $^{***}$p<0.01 |

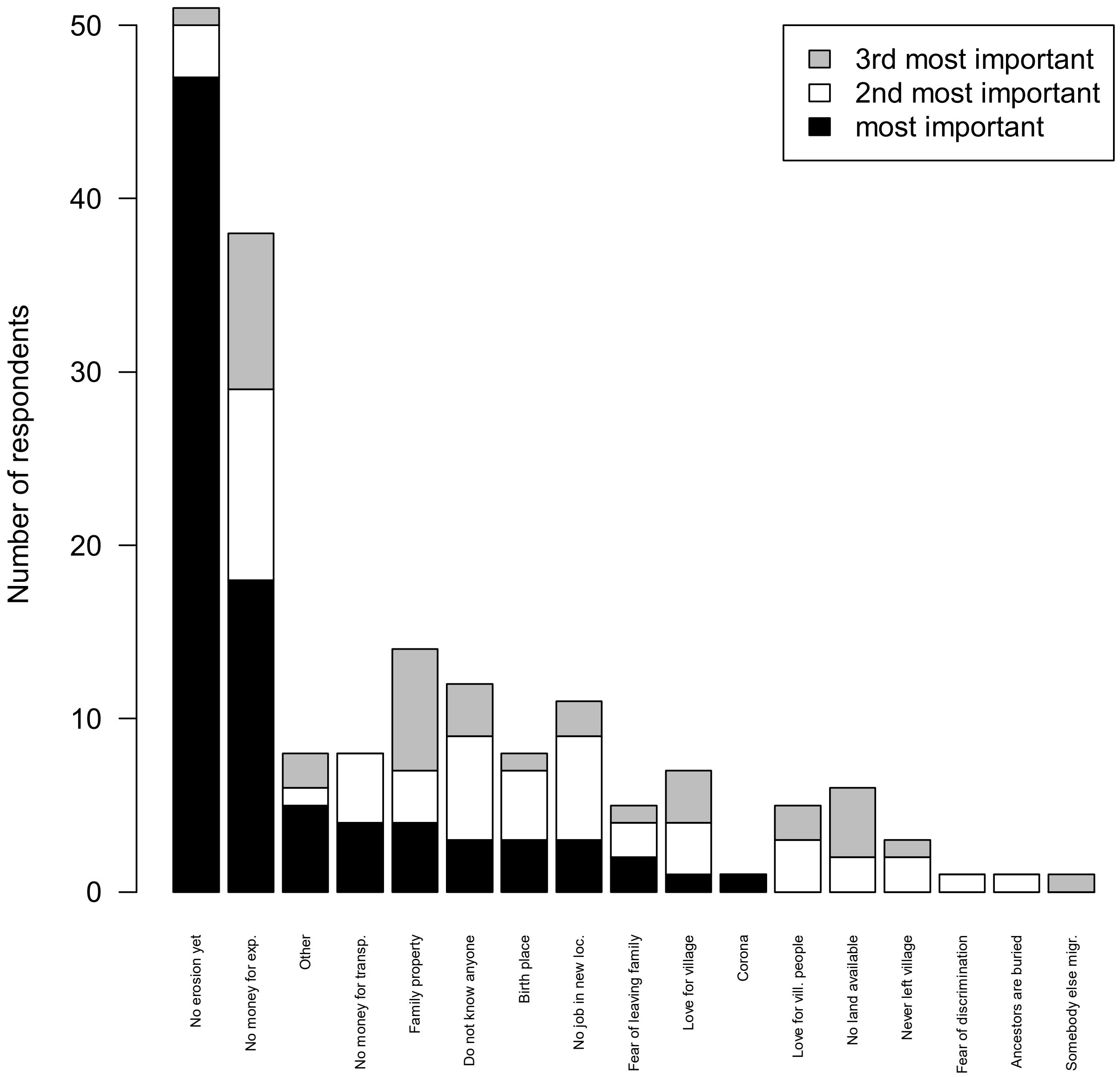


Fig. S 6: Self-reported reasons for non-migration among those respondents who had previously considered moving away.

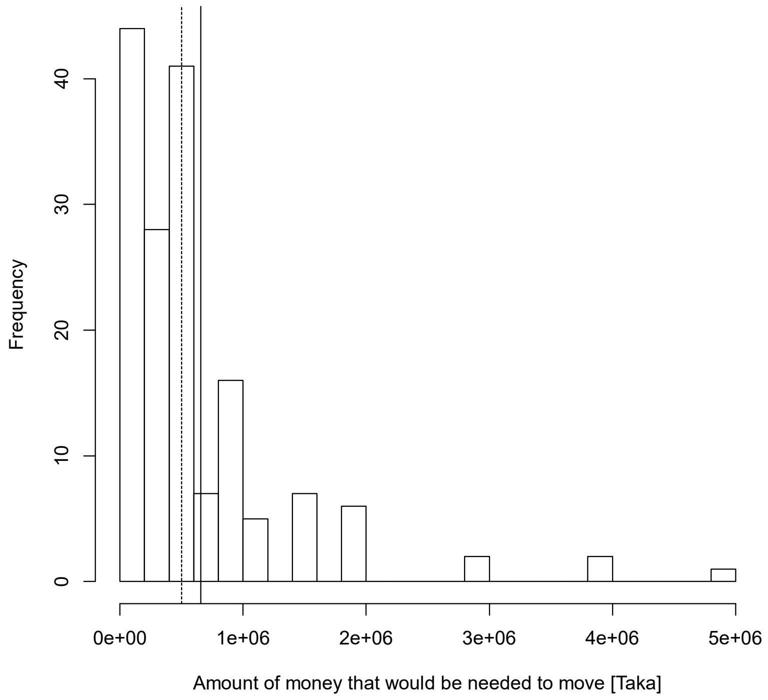


Fig. S 7: Amount of money needed by resource-constrained respondents to undertake the aspired move. Vertical lines: median (dashed) and mean (solid). 1 Taka ~ 0.01 USD (as of January 2022).

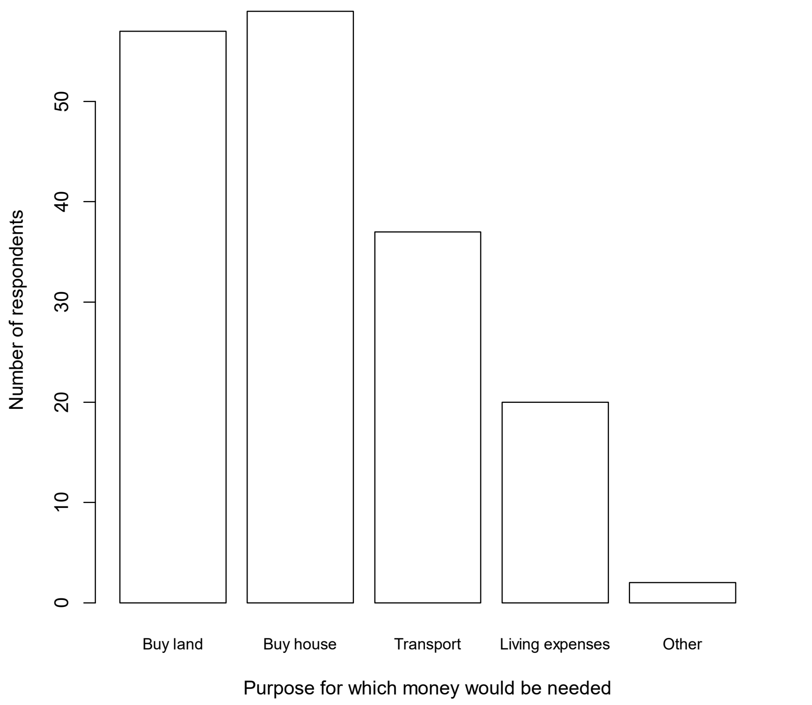


Fig. S 8: Purpose for which resource-constrained respondents would need money to undertake the aspired move (multiple answers possible).

# Appendix D: Pre-registration

## D.1 Anonymized version of pre-analysis plan

Even if two individuals or households are exposed to the same environmental/climatic event, they do not necessarily react the same way: One might migrate, while the other might stay. In this project, we examine the micro-level processes connecting the exposure to an environmental event and a migration decision. Individuals will only migrate if they have both an aspiration and the ability to move (Carling, 2002; Carling & Schewel, 2018). Conversely, this implies that there might be individuals who would like to migrate, but cannot (involuntary non-migrants or trapped populations) as well as others who could migrate, but do not want to, even though they might face a physical need to move (voluntary non-migrants). This project seeks to distinguish these three groups (migrants being the third group) by examining the relationship of migration aspirations and the ability to move.

Thereby, we define “migration aspirations” as “a person’s thoughts and feelings about the prospect of changing their place of residence” (Carling, 2019, p. 8). We differentiate these subjective aspirations from an objective “need to move”, which we define as a necessity to move since an individual’s/household’s livelihood comes under pressure, since it is no longer sustainable in the current location. Differentiating aspirations and need is important to fully understand micro level decision processes. Black et al. (2013), for instance, only consider vulnerability to extreme events and ability to move to infer the (conceptual) existence of trapped populations. This concept lacks, however, the individuals’ aspirations, which are crucial for the definition of trapped populations. Therefore, we need to understand what contributes to migration aspirations:

> *RQ1: How are migration aspirations influenced by exposure to an environmental event and how do they relate to actual migration moves?*

Migration aspirations are influenced by a number of factors, which we include as control variables into the following analyses. One important factor is an individual’s attachment to place. Adams and Adger (2013) argue that environmental factors enter the migration decision-making process through their contribution to place utility, which they define as a function of both affective and instrumental bonds to location. Further, Adams (2016) shows that place attachment can have a stronger effect on immobility than resource constraints. Other

mediating factors that we will include are faith in God (where we expect more religious individuals to have a lower desire to move since they perceive reduced agency over their life) and risk preference (where we expect risk averse individuals to have a lower desire to move since the risk of moving is perceived as higher than the risk of staying, following Carling 2002).

Following Black et al. (2013), we proxy a household's need to move by their vulnerability to a certain environmental event. Vulnerability is formed by three components, namely exposure, sensitivity and adaptive capacity (IPCC, 2007). We define exposure as the perceived or actual (direct or indirect) impact of environmental change on the livelihood of the household. Sensitivity is the degree to which a system is affected by or responsive to environmental stimuli, whereas adaptive capacity describes the ability to prepare for, respond to and tackle the effects of environmental change. Since in our research design, exogenous variation occurs only in terms of exposure, this is our independent variable for the subsequent hypotheses (alternatively, we might use proximity to the riverbank as an instrument), whereas we take sensitivity and adaptive capacities into account by controlling for them.

We plan to measure migration aspirations in each of the four rounds of the panel. In the following, we describe our hypotheses and related analyses for two rounds for reasons of simplicity. The reasoning can, however, be extended to all four rounds. In the baseline survey before the monsoon, we measure migration aspirations for all respondents. During the monsoon, a part of the sample will be exposed to environmental events (mainly riverbank erosion and/or floods), while the other part will not be exposed. Further, a part of the sample will migrate during or after the monsoon (either as an entire household or individually), while the other part will not migrate. When we conduct the second round of interviews after the monsoon, we are thus faced with four groups (Table S 12).

Table S 12: Respondents of the first follow-up survey fall into one of four categories.

| | Has migrated | Has not migrated |
|---|---|---|
| Has been exposed | 1 | 2 |
| Has not been exposed | 3 | 4 |

These four groups allow for different analyses related to migration aspirations. First, we will compare those who have migrated (groups 1+3) with those who have not migrated (groups 2+4) in terms of their migration aspirations during the baseline survey. Tjaden, Auer, and Laczko (2019) have shown a strong association between migration intentions and actual migration flows. If this holds true, measuring migration aspirations could serve as a proxy to predict migration flows. Therefore, we test the following hypothesis:

> *H1: The higher the migration aspiration before the monsoon, the higher the likelihood of migrating during or after the monsoon, controlling for ability to move.*

Second, we will re-assess the migration aspirations after the monsoon for those who have not migrated (groups 2+4). Exposure corresponds to a livelihood threatening loss of assets or land. Therefore, we expect those who have been exposed to an environmental event (group 2) to have higher migration aspirations after the monsoon season than before. Further, migration aspirations are not a stable character trait, but are highly situational and can be volatile (Carling 2019, p. 14). We would expect the overall livelihood situation in the study area to be more challenging immediately after the monsoon (e.g. due to flood-related infrastructure damages or the exposure of close relatives or friends, even if the own household has not been exposed). Accordingly, we expect migration aspirations to be higher after the monsoon than before also for those respondents who have not been exposed themselves. We thus expect an overall increase in migration aspirations among all respondents who have not migrated, but this increase should be stronger among those who have been exposed than among those who have not been exposed. We plan to analyze this effect in a difference-in-difference design.

> *H2: Average migration aspirations are higher after than before the monsoon.*

> *H3: Migration aspirations increase more for respondents who have been exposed than for those who have not.*

Third, we will focus in more detail on the characterization of those who have not migrated (groups 2+4). It is important to differentiate whether their non-migration is because they have no migration aspirations in the first place (voluntary non-migrants), or because they have aspirations, but lack the ability to move (involuntary non-migrants): If households do not have enough resources to migrate, they will stay *in situ* despite potential migration aspirations. This is the basic idea behind the term "trapped populations". While the term has seen a

lot of conceptual discussion in recent literature (cf. Ayeb-Karlsson et al., 2018 for a discursive review), little empirical work has been done investigating the extent and nature of trapped populations:

*RQ2: How many of those who did not migrate are voluntary non-migrants, and how many are involuntary non-migrants?*

Irrespective of whether they are voluntary or involuntary non-migrants, those who have been exposed (group 2) have to cope with the effect of the exposure on their livelihood *in situ*, given that they have not migrated. If policy makers wish to support these parts of the population, they need to understand these coping strategies. Also, they need to understand the obstacles preventing those who have migration aspirations from actually moving. Therefore, we ask the exploratory questions:

*RQ3: What are in situ coping strategies of those respondents who are exposed to environmental change, but do not migrate?*

*RQ4: Which factors prevent involuntary non-migrants from migrating?*

Also, policy makers require information on the socio-demographic composition of the three groups (migrants as well as voluntary and non-voluntary non-migrants). The ability to move is correlated to a household's access to resources, which is, for instance, lower for poor and female headed households (Akter et al., 2019; Alam, 2017).

*RQ5: What is the socio-demographic composition of migrants, voluntary non-migrants and involuntary non-migrants, differentiated by exposure?*

To analyze these questions, we mostly use data from the household surveys. Specifically, we will use the following constructs:

- Exposure – determined from the perception of the environmental event and/or the affectedness by the event (depending on the results of section X).
- Sensitivity – determined from occupation, land/house ownership, financial resources (income, credit, savings, and meals per day) as well as the diversity of livelihood strategies.

- Adaptive capacities – determined from adaptation strategies applied for past erosion/flood events, education (as a proxy for human capital following van der Land & Hummel, 2013), wealth (as a proxy for financial capital) and social networks.
- Attachment to place – determined from length of residence in present location, home ownership, and a self-assessment.
- Faith in god – determined from questions about religious practices
- Risk preference – determined from a self-assessment
- Desire to move – determined from present aspirations to move, their choice in a hypothetical decision scenario, and whether they had thought about migrating in the past, following Carling & Schewel (2018) and Carling (2019).
- Ability to move – determined from the respondents' financial resources (physical capital) as well as from their social network and whether they or other members of the household have previously migrated (social capital), following Wiederkehr et al. (2019, p. 7).
- Migration decisions – derived from the post-monsoon survey as well as from tracking the respondents during the monsoon season.

## D.2 Modifications to the pre-analysis plan

I partially adapted the pre-analysis plan and report the differences here for reasons of transparency.

- Scope 1: Initially, this subproject was conceived to study both the link from environmental exposure to migration aspirations, and from aspirations to actual moves. To narrow the focus of the paper, it focuses only on the link from migration aspirations to actual moves. The link from environmental exposure to migration aspirations is examined in another subproject / paper.
- Scope 2: Likewise, the temporal evolution of migration aspirations (H2 and H3) is studied in a separate project.

# References


Adams, H. (2016). Why populations persist: Mobility, place attachment and climate change. *Population and Environment*, *37*(4), 429–448. https://doi.org/10.1007/s11111-015-0246-3

Adams, H., & Adger, N. W. (2013). The contribution of ecosystem services to place utility as a determinant of migration decision-making. *Environmental Research Letters*, *8*(1), 015006. https://doi.org/10.1088/1748-9326/8/1/015006

Akter, K., Dey, S., & Hasan, S. (2019). Riverbank erosion and its impact on rural women: Case study of Ulania village in Bangladesh. *Asian Journal of Women's Studies*, *25*(1), 76–95. https://doi.org/10.1080/12259276.2019.1577343

Alam, G. M. M. (2017). Livelihood Cycle and Vulnerability of Rural Households to Climate Change and Hazards in Bangladesh. *Environmental Management*, *59*(5), 777–791. https://doi.org/10.1007/s00267-017-0826-3

Alam, G. M. M., Alam, K., Mushtaq, S., & Clarke, M. L. (2017). Vulnerability to climatic change in riparian char and river-bank households in Bangladesh: Implication for policy, livelihoods and social development. *Ecological Indicators*, *72*, 23–32. https://doi.org/10.1016/j.ecolind.2016.06.045

Ayeb-Karlsson, S., Smith, C. D., & Kniveton, D. (2018). A discursive review of the textual use of 'trapped' in environmental migration studies: The conceptual birth and troubled teenage years of trapped populations. *Ambio*, *47*(5), 557–573. https://doi.org/10.1007/s13280-017-1007-6

Black, R., Arnell, N. W., Adger, W. N., Thomas, D., & Geddes, A. (2013). Migration, immobility and displacement outcomes following extreme events. *Environmental Science & Policy*, *27*, 32–43. https://doi.org/10.1016/j.envsci.2012.09.001

Carling, J. (2002). Migration in the age of involuntary immobility: Theoretical reflections and Cape Verdean experiences. *Journal of Ethnic and Migration Studies*, *28*(1), 5–42. https://doi.org/10.1080/13691830120103912

Carling, J. (2019). *Measuring migration aspirations and related concepts: MIGNEX Background Paper*.

Carling, J., & Schewel, K. (2018). Revisiting aspiration and ability in international migration. *Journal of Ethnic and Migration Studies*, *44*(6), 945–963. https://doi.org/10.1080/1369183X.2017.1384146

CEGIS. (2018). *Update, improve and extend the erosion forecasting and warning tools in the three main rivers*.

Crawford, T. W., Rahman, M. K., Miah, Md. G., Islam, Md. R., Paul, B. K., Curtis, S., & Islam, Md. S. (2020). Coupled Adaptive Cycles of Shoreline Change and Households in Deltaic Bangladesh: Analysis of a 30-Year Shoreline Change Record and Recent Population Impacts. *Annals of the American Association of Geographers*, 1–23. https://doi.org/10.1080/24694452.2020.1799746

Freihardt, J., & Frey, O. (2023). Assessing riverbank erosion in Bangladesh using time series of Sentinel-1 radar imagery in the Google Earth Engine. *Natural Hazards and Earth System Science*, *23*(2), 751–770. https://doi.org/10.5194/nhess-23-751-2023

IPCC. (2007). *Climate change 2007—Impacts, adaptation and vulnerability: Contribution of Working Group II to the Fourth Assessment Report of the Intergovernmental Panel on Climate Change* (1. publ). Cambridge Univ. Press. http://www.ipcc.ch/ipccreports/ar4-wg2.htm

Islam, M. S., Sultana, S., Saifunnahar, & Miah, M. A. (2015). Adaptation of Char Livelihood in Flood and River Erosion Areas through Indigenous Practice: A Study on Bhuapur Riverine Area in Tangail. *Journal of Environmental Science and Natural Resources*, *7*(1). https://doi.org/10.3329/jesnr.v7i1.22138

Sarker, M. H., Thorne, C. R., Aktar, M. N., & Ferdous, Md. R. (2014). Morpho-dynamics of the Brahmaputra–Jamuna River, Bangladesh. *Geomorphology*, *215*, 45–59. https://doi.org/10.1016/j.geomorph.2013.07.025

Tjaden, J., Auer, D., & Laczko, F. (2019). Linking Migration Intentions with Flows: Evidence and Potential Use. *International Migration*, *57*(1), 36–57. https://doi.org/10.1111/imig.12502

van der Land, V., & Hummel, D. (2013). Vulnerability and the Role of Education in Environmentally Induced Migration in Mali and Senegal. *Ecology and Society*, *18*(4). https://doi.org/10.5751/ES-05830-180414

Wiederkehr, C., Schröter, M., Adams, H., Seppelt, R., & Hermans, K. (2019). How does nature contribute to human mobility? A conceptual framework and qualitative analysis. *Ecology and Society*, *24*(4). https://doi.org/10.5751/ES-11318-240431
